# Associations of selenium status with all-cause and cause-specific mortality: a systematic review and meta-analysis of cohort studies

**DOI:** 10.1016/j.redox.2025.103755

**Published:** 2025-07-10

**Authors:** Zhixin Cui, Ruijie Xie, Xiaoting Lu, Lutz Schomburg, Hermann Brenner, Ben Schöttker

**Affiliations:** a Division of Clinical Epidemiology and Aging Research, German Cancer Research Center, Im Neuenheimer Feld 581, 69120 Heidelberg, Germany; b Medical Faculty Heidelberg, Heidelberg University, Im Neuenheimer Feld 672, 69120 Heidelberg, Germany; c Department of Clinical Nutrition, Sun Yat-sen Memorial Hospital, Sun Yat-sen University, Yanjiang West Road 107, 510120 Guangzhou, China; d Institute of Experimental Endocrinology, Max Rubner Center (MRC) for Cardiovascular Metabolic Renal Research, Charité University Medicine Berlin, CCM, Hessische Straße 4A, 10115 Berlin, Germany

**Keywords:** Systematic review, Meta-analysis, Selenium, Mortality, Cardiovascular diseases, Cancer, Cohort studies

## Abstract

**Objective:**

To provide a systematic review and meta-analysis of population-based cohort studies on the association of selenium status with all-cause and cause-specific mortality.

**Methods:**

Relevant studies were identified through systematic searches of MEDLINE and ISI Web of Knowledge. Risk ratios (RRs) reported across categories of selenium biomarkers were recalculated as continuous RR estimations per standard deviation (SD) using generalized least squares for linear trend estimation and pooled in random effects meta-analyses.

**Results:**

The literature search identified 20 studies, including 17 studies on all-cause mortality, 9 studies on cardiovascular mortality and 7 on cancer mortality. An increase of selenium biomarker concentration by one SD was associated with 13 % lower all-cause mortality (RR [95 %-confidence interval], 0.87 [0.83–0.90]), 11 % lower cardiovascular mortality (0.89 [0.84–0.94]) and 15 % lower cancer mortality (0.85 [0.78–0.94]). Although moderate heterogeneity was observed, the inverse association with all-cause mortality was robust across countries with low or adequate selenium supply, selenium measurement methods, recruitment years, study quality scores, follow-up lengths and sample sizes. The trim and fill method showed no indications of relevant publication bias.

**Conclusion:**

Selenium biomarkers are inversely associated with all-cause, cardiovascular and cancer mortality in the general population and clinical trials among selenium deficient populations are still needed.

## 1 Introduction

Selenium (Se) is an essential trace element for human health. Se content in soil varies broadly throughout the world [1]. Consequently, dietary intake of Se and resulting Se status also varies from high to low based on geography [1,2]. The Se status can be measured as Se concentration in whole blood, plasma/serum and nail/hair [3]. Furthermore, it can also be determined by measuring the circulating concentration of selenoprotein P (SELENOP) or the enzyme activity of glutathione peroxidases (GPXs), which are selenoproteins as well [3].

Low Se status leads to poor expression of selenoproteins [4]. Selenoproteins are composed of 25 members, and perform a wide range of physiological functions [[5], [6], [7]]. GPXs and thioredoxin reductases (TXNRDs) are involved in antioxidant protection and redox functions. GPXs catalyze the removal of hydrogen peroxide and lipid hydroperoxides, protecting vessels from damage caused by oxidative stress and inflammation. TXNRDs play a significant role in the redox regulation of metabolism and cellular functions [[6], [7], [8]]. According to the free radical/oxidative stress theory of aging, reactive oxygen species (ROS) accumulation, oxidative stress, and imbalance of the normal redox state increase with age, which leads to cumulative DNA, protein, and lipid damage, and thereby increases the risk of cardiovascular disease (CVD), cancer and death [[9], [10], [11], [12]]. Therefore, suboptimal Se status may have an impact on premature death.

Some epidemiological studies reported inverse associations of Se status with all-cause mortality [13,14], CVD mortality [15,16] and cancer mortality [13,17], but these associations were not observed with statistical significance in other studies [[18], [19], [20]]. These heterogeneous findings call for critical evaluation in a comprehensive systematic review, that judges the quality of the studies and provides meta-analyses. However, previous systematic reviews and meta-analyses on these mortality endpoints had a variety of limitations [[21], [22], [23]]. They did not include all available studies for one outcome in one meta-analysis but presented distinct meta-analyses by comparing the lowest and highest category of circulating Se and then provided risk estimates per 1 standard deviation (SD) increase in Se concentration. The meta-analyses using the categories are especially difficult to interpret, because the cut-offs to define the lowest and highest category varied largely across the studies (e.g., quintiles, quartiles, and tertiles). Moreover, some recent publications of large cohort studies were not included [[13], [14], [15], [16],^18^,^19^].

Thus, we conducted a new systematic review with meta-analyses of all available studies applying linear trend estimation for the association of circulating Se status with all-cause mortality, CVD mortality and cancer mortality in population-based cohort studies.

## 2 Methods

The protocol of this study was registered in PROSPERO [CRD42023420652] and is being reported according to the Meta-analysis Of Observational Studies in Epidemiology (MOOSE) statement [24] (Appendix Table 1).

### 2.1 Search strategy

A systematic literature search was carried out in the databases MEDLINE (Ovid Technologies, New York) via PubMed and ISI Web of Knowledge (Thomson Scientific Technical Support, New York) up to March 11, 2025. We combined synonymous or related terms for the study exposure (selenium) and the outcomes (mortality) (see Appendix Tables 2 and 3 for search terms in PubMed and ISI Web of Knowledge, respectively). Studies with the publication type classified as “randomized controlled trial”, “review”, “editorial”, “letter” “meeting”, “patent”, “book”, “case report” or “news” were excluded. There were no restrictions on publication language or date. The reference manager Endnote X9 (Clarivate Analytics, Philadelphia, PA) was used throughout the literature search and screening process.

### 2.2 Eligibility criteria and data extraction

Duplicate publications identified in both MEDLINE and ISI Web of Knowledge were excluded. The title and abstract of the remaining studies were reviewed to evaluate if they were relevant to the topic.

The full-text review was independently conducted by two reviewers (ZC and RX). Studies were excluded if they met any of the following exclusion criteria: a) no cohort study design; b) lack of Se status measurement, such as serum/plasma concentration of Se, GPXs or SELENOP; c) no assessment of the association between Se status with all-cause mortality, CVD mortality or cancer mortality; d) with participants under 18 years of age, pregnant or lactating women, or participants who were critically ill or had serious chronic infections; e) no reported relative risk estimates suitable for meta-analysis; f) duplicate publications from the same study population (only the publication with the highest number of cases was included). For studies that only reported risk estimates across categories of Se concentration, we further excluded those that did not report either the cut-off values or the case number of each Se category, as these were necessary parameters for linear trend estimation using the “dosresmeta” R package [25]. We wrote to the corresponding authors of these publications to retrieve the missing data [[26], [27], [28], [29]] but no response was received. Finally, references of the included studies were screened (cross-referencing) to ensure that the literature search was comprehensive.

Data from each included study were independently extracted by two reviewers (ZC and XL) using predetermined data extraction forms. If an included study reported multiple risk estimates, we extracted the risk estimate that was most comprehensively adjusted for confounding factors. Consensus was reached through discussion or consulting a third reviewer (BS).

### 2.3 Assessment of study quality

The modified Newcastle-Ottawa scale (NOS) for cohort studies was used to assess the quality of the included studies and its adaption for this review is shown in Appendix Table 4 [30,31].

### 2.4 Data synthesis and statistical analysis

The R software was used for all analyses (version 4.1.3, The R Foundation for Statistical Computing, Vienna, Austria). For consistency, all circulating Se concentrations were converted to μg/L. For example, 1 μg/L = 1 μmol/L × molecular weight of Se, and the molecular weight of Se is 78.96 g/mol [32]. Given the various categorizations of Se concentrations across studies, we employed generalized least squares for trend estimation using the “dosresmeta” package [25,33], under the assumption of a linear dose-response relationship between the Se concentration and the outcomes of interest [21]. For the trend estimation, it was essential to obtain the number of cases, the number of participants or person-years, the reported risk ratios (RRs), and the median value of Se/SELENOP concentration for each category [25]. For studies, which did not report median values for each category, we estimated the median for the middle categories by calculating the midpoint of the lower and upper borders [34]. For the lowest and highest Se/SELENOP categories, we estimated the medians by assuming a normal distribution of Se/SELENOP concentration based on the mean and SD [21]. Using the midpoint method would have led to medians estimated too low for the lowest Se/SELENOP category and too high for the highest Se/SELENOP, leading to a wider Se/SELENOP distribution and a falsely high weight for the study in the meta-analysis. In practice, for studies using quintiles for example, we used the z-statistic to estimate the 10th percentile (P_10_) and 90th percentile (P_90_) as the missing medians of the lowest and highest quintiles with the following equation:

$$P_{10}=\text{mean}+Z_{P10}\;x\;\text{SD}$$

$$P_{90}=\text{mean}+Z_{P90}\;x\;\text{SD}$$

Z_P10_ and Z_P90_ are the z-statistics calculated by using “qnorm()” in R software.

We observed that, compared to the midpoint method, with this method, the weight of each study in the random effects meta-analysis was closer to the weight each study had in a simple fixed effects meta-analysis weighting the studies by case numbers. However, we made one exception. For the study of Kok et al. [35], the midpoint method for all categories provided a study weight closer to the weight in the fixed effects meta-analysis weighted by case numbers, and thus exceptionally, this method was used for Kok et al. [35].

If risk estimates were reported for a specific increment increase (e.g. 10 μg/L), the RR per one SD increase was calculated by the following equation:

$${\text{RR}}_{\text{per}\;\text{SD}\;\text{increase}}={\left({\text{RR}}_{\text{per}\;10\;\text{unit}\;\text{increase}}\right)}^{\text{SD}/10}$$

The equation was also suitable for calculating the lower and upper 95 % confidence interval (CI) per SD increase.

For studies, which reported an RR per SD decrease, we changed the direction to RR per SD increase with the following equation:

$${\text{RR}}_{\text{per}\;\text{SD}\;\text{increase}}=1/\;{\text{RR}}_{\text{per}\;\text{SD}\;\text{decrease}}$$

The same equation was applied to re-calculate the 95 % CIs.

For studies without a reported SD but reported quartiles (with 25^th^ percentile (P_25_) and 75^th^ percentile (P_75_)) or quintiles (with 20^th^ percentile (P_20_) and 80^th^ percentile (P_80_)), we converted the assumed normal distribution of circulating Se into standard normal distribution, and estimated the SD by the following equations:

$$\text{SD}\approx \left(P_{75}\;–\;P_{25}\right)/\left(Z_{P75}\;–\;Z_{P25}\right)$$

$$\text{SD}\approx \left(P_{80}\;–\;P_{20}\right)/\left(Z_{P80}\;–\;Z_{P20}\right)$$

Z_P20_, Z_P25_, Z_P75_ and Z_P80_ are the z-statistics at specific Se/SELENOP percentiles (20^th^, 25^th^, 75^th^, and 80^th^ percentile, respectively), calculated by using “qnorm()” in R software.

For studies without a reported SD and arbitrary Se biomarker cut-offs (study A), we estimated the SD from the mean and SD from another study B by the following equation:

SD(studyA)=MeanSe(studyA)xSD(studyB)/mean(studyB)

This equation was only used if studies A and B had a similar mean Se concentration and the same Se measurement method.

We conducted random effects meta-analyses to estimate summary RRs and 95 % CIs per SD increment of Se/SELENOP concentration. Cochran's Q test and the *I*^*2*^ statistic were performed for heterogeneity among studies and interpreted as not important, moderate, substantial or considerable according to the Cochrane Handbook [36]. Publication bias was assessed using Egger's tests and funnel plots [37]. In cases of detected publication bias, the trim and fill method was applied to impute the results of potentially non-published studies [38]. In sensitivity analyses, we checked the robustness of the pooled effect estimates by excluding each single study one by one. Subgroup analyses were performed stratified by region (Europe, Asia, or Northern America), countries with low or adequate Se supply (low Se supply was defined by measured mean plasma Se < 100 μg/L in most previous studies from the respective country as summarised in the review of Combs et al. [2]), measurement methods for Se biomarkers (atomic absorption spectrometry (AAS) for plasma/serum Se, inductively coupled plasma mass spectrometry (ICP-MS) for plasma/serum Se or enzyme-linked immunosorbent assay (ELISA) for SELENOP), recruitment years of the studies (<1998 or ≥1998), duration of follow-up (<10 years or ≥10 years), sample size (<3000 or ≥ 3000 participants), and study quality (NOS score <8 or NOS score ≥8).

## 3 Results

### 3.1 Literature search

**Fig. 1:**
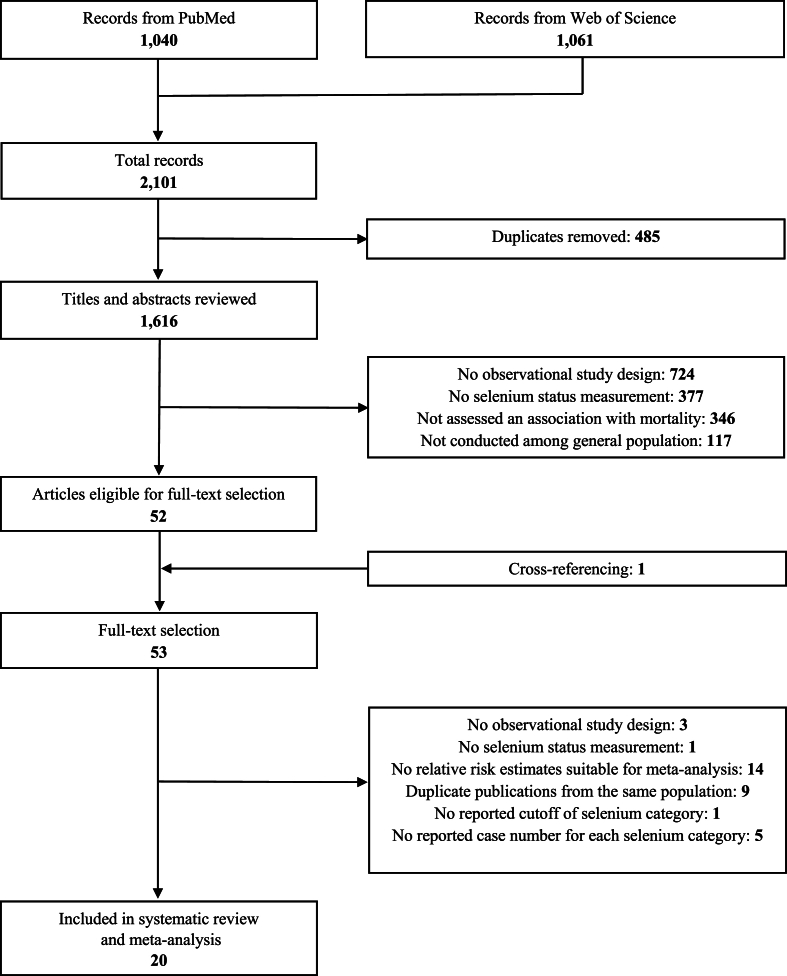
Flow chart of literature search.

The process of literature search is shown in Fig. 1. We identified 2101 publications after excluding 485 duplicates. Of those, 1616 publications met the exclusion criteria during title and abstract review, leaving 52 articles. As 1 additional study was identified through cross-referencing, there were 53 studies for full-text review. We excluded 33 studies that met the exclusion criteria and listed the references in Appendix Tables 5 and 20 studies with a total of 67,534 participants were subsequently included in this systematic review.

### 3.2 Description of included studies

Details of the 20 studies are shown in Table 1. The studies were published between 1987 and 2024. Thirteen studies originated from Europe, 5 were conducted in Northern America, and 2 studies were carried out in Asia. The follow-up time ranged from 4.3 years to 25.7 years. Eighteen studies measured serum/plasma Se as the biomarker of Se status and 2 measured serum/plasma SELENOP. We did not identify studies using other biomarkers of Se status. Two studies were conducted only in males and one only in females, whereas 17 studies included participants of both sexes. Thirteen out of the 20 studies adjusted or stratified for the key set of covariates (Age, sex (if not restricted to one sex), body mass index or equivalent, and smoking).

**Table 1:** Details of studies included in the meta-analyses on the associations of selenium status with all-cause mortality, cardiovascular mortality and/or cancer mortality.

First author, publication year, study acronym	Country (baseline) follow-up (years)	Age (years)^a^ (sex)	N_total_^b^	Se method	Se concentration^c^ (μg/L for Se, mg/L for SELENOP)	All-cause mortality		Cardiovascular mortality		Cancer mortality		Key set of covariates and stratification factors^e^
						N_death_	RR (95 % CI)^d^	N_death_	RR (95 % CI)^d^	N_death_	RR (95 % CI)^d^	
Kok, 1987 EPOZ	Netherlands (1975–1978) 6–9	37 - 87 (both)	252	serum Se	126.0 ± 28.6^g^ <105.0 105.0–116.9 117.0–129.6 129.7–153.0 >153.0	–	–	84	2.0 (0.8; 5.0) 1.7 (0.6; 4.4) 0.7 (0.3; 2.0) 1.8 (0.7; 4.3) 1	–	–	Yes^l^
Kok, 1987 EPOZ	Netherlands (1975–1978) 6–9	60.0 (mean) (both)	207	serum Se	125.9 ± 28.5^h^ <102.8 ≥102.8	–	–	–	–	69	1.9 (0.9; 4.0) 1	Yes^m^
Kilander, 2001 –	Sweden (1970–1973) 25.7 (maximum)	48.6–51.1 (male)	2285	serum Se	75.9 (mean)	630	0.87 (0.80; 0.95) per SD increase	301	0.97 (0.84; 1.12) per SD increase	216	1.02 (0.87; 1.20) per SD increase	No, not for BMI and smoking
Wei, 2004 –	China (1985) 15	56.6 ± 8.0 (both)	1103	serum Se	73.4 ± 10.1^h^	516	0.96 (0.90; 1.02) per 11.5 μg/L increase	–	–	–	–	Yes^n^
Akbaraly, 2005 EVA	France (1991–1993) 9	65.0 (mean) (both)	1290	plasma Se	86.3 ± 15.8	101	1.54 (1.25; 1.88) per SD decrease	–	–	45	1.79 (1.32; 2.44) per SD decrease	Yes^o^
Walston, 2006 WHAS I	USA 1992 5	77.3 ± 7.8 (female)	591	serum Se	118.2 ± 19.2 ≤109.9 109.9–122.8 >122.8	197	1.54 (1.03; 2.32) 1.30 (0.86; 1.96) 1	–	–	–	–	Yes^p^
González, 2007 –	Spain (1999–2002) 4.3 (mean)	75.1 ± 6.5 (both)	215	serum Se	86.9 ± 17.4 ≤72.64 73.43–82.12 82.91-90.01 90.80–98.70 >99.49	60	1 1.13 (0.54; 2.40) 0.69 (0.29; 1.60) 0.81 (0.36; 1.82) 0.57 (0.23; 1.40)	–	–	–	–	Yes^q^
Lauretani, 2008 InCHIANTI	Italy (1998–2000) 6	75.6 ± 7.4 (both)	1042	plasma Se	74.2 ± 12.6	237	0.32 (0.13; 0.79) per 1 μmol/L increase	–	–	–	–	No, not for smoking^r^
Bates, 2011 BNDNS	UK (1994–1995) 13–14	76.6 ± 7.4 (both)	629	plasma Se	73.0 ± 16.7 ( × 10^−3^)^i^	403	0.83 (0.73; 0.94) per SD increase	105	0.84 (0.67; 1.06) per SD increase	87	0.78 (0.60; 1.01) per SD increase	Yes^s^
Suadicani, 2012 CMS	Denmark (1985–1986) 16	62.9 (mean) (male)	3333	serum Se	93.9 ± 19.2^h^ 31.58–78.96 86.86–94.75 102.65–236.88	1429	1 0.83 (0.73; 0.95) 0.90 (0.79; 1.03)	–	–	–	–	No, not for BMI and smoking^t^
Goyal, 2013 NHANES III	USA (1988–1994) 14.2 (median)	≥20 (both)	14,727^f^	serum Se	125.5 ± 17.3 ( × 10^−3^)^h^ ≤108.96 ( × 10^−3^) 110.54–118.44 ( × 10^−3^) 119.23–126.34 ( × 10^−3^) 127.13–135.02 ( × 10^−3^) ≥136.60 ( × 10^−3^)	4086	1 0.74 (0.63; 0.87) 0.77 (0.66; 0.91) 0.69 (0.56; 0.85) 0.79 (0.68; 0.92)	1825	1 0.76 (0.61; 0.95) 0.77 (0.63; 0.96) 0.89 (0.71; 1.12) 0.83 (0.67; 1.04)	864	1 0.76 (0.52; 1.12) 0.81 (0.62; 1.08) 0.53 (0.36; 0.79) 0.86 (0.62; 1.20)	Yes^u^
Alehagen, 2016 –	Sweden (2003) 6.85	77.7 (mean) (both)	449	serum Se	67.1 ± 16.8 <57.2 ≥57.2	122	1.43 (1.02; 2.00) 1	85	1.56 (1.03; 2.36) 1	–	–	No, not for age and BMI^v^
Giovannini, 2018 ilSIRENTE	Italy (2003–2004) 10	85.8 (mean) (both)	347	serum Se	105.3^j^ ± 20.4^h^ ≤105.3 >105.3	248	1 0.71 (0.54; 0.92)	–	–	–	–	No, not for smoking^w^
Schomburg, 2019 MPP	Sweden (2002–2006) 9.3 (median)	69.4 (mean) (both)	4366	plasma SELENOP	5.5^j^ ± 1.6^h^ 0.4–4.3 4.3–5.1 5.1–5.9 5.9-6.9 6.9–20	1111	1 0.73 (0.61; 0.87) 0.66 (0.55; 0.79) 0.57 (0.48; 0.69) 0.69 (0.58; 0.82)	351	1 0.65 (0.48; 0.89) 0.66 (0.48; 0.89) 0.52 (0.37; 0.72) 0.59 (0.43; 0.81)	–	–	Yes^x^
Li, 2020 NHANES 1999–2006	USA (1999–2006) 10.2 (median)	61.9 ± 13.7 (both)	2896	serum Se	136.4 ± 19.6 ≤124.0 125.0–135.0 136.0–147.0 ≥148.0	858	1 0.62 (0.47; 0.81) 0.57 (0.42; 0.75) 0.60 (0.45; 0.78)	–	–	–	–	Yes^y^
Shi, 2021 DF-TJ	China (2008–2010) 9.8 (mean)	64.9 ± 7.5 (both)	6155	plasma Se	65.3^j^ ± 14.6^k^ <56.44 56.44–65.32 65.32–76.12 ≥76.12	876	1 1.02 (0.85; 1.22) 0.81 (0.67; 0.98) 0.68 (0.56; 0.83)	416	1 1.05 (0.80; 1.37) 0.85 (0.64; 1.12) 0.67 (0.50; 0.89)	–	–	Yes^z^
Al-Mubarak, 2022 PREVEND	Netherlands (2001–2003) 8.4 (median)	53.6 ± 12.1 (both)	5973	serum Se	84.6 ± 19.5	381	0.95 (0.87; 1.04) per 10 μg/L increase	–	–	–	–	Yes{
Schöttker, 2024 ESTHER	Germany (2000–2002) 17.3 (median)	62.3 ± 6.6 (both)	7186	serum SELENOP	4.8 ± 1.4	2126	0.89 (0.85; 0.93) per SD increase	709	0.93 (0.86; 1.01) per SD increase	696	0.90 (0.83; 0.97) per SD increase	Yes|
Li, 2024 NHANES 2011–2018	USA (2011–2018) 4.3 (median)	≥20 (both)	8989	serum Se	192.96^j^ ± 27.61^h^ <192.96 ≥192.96	861	1 0.70 (0.58; 0.84)	–	–	–	–	No, not for smoking}
Jiang, 2024 NHANES 2003–2004, 2011–2016	USA (2003–2004, 2011–2016) –	48.4 ± 15.8 (both)	5499	serum Se	130.5^j^ ± 16.4^k^ <119.9 119.9–130.4 130.5–1420.0 142.1–313.0	–	-	252	1 0.63 (0.36; 1.11) 0.49 (0.32; 0.76) 0.69 (0.43; 1.10)	179	1 0.53 (0.29; 0.97) 0.65 (0.33; 1.27) 0.52 (0.29; 0.92)	No, not for BMI∼

Abbreviations: BMI, body mass index; BNDNS, the British National Diet and Nutrition Survey; CI, confidence interval; CMS, the Copenhagen Male Study; CRP, C-reactive protein; CVD, cardiovascular disease; DF-TJ, the Dongfeng-Tongji cohort; eGFR, epidermal growth factor receptor; ESTHER, the Epidemiologische Studie zu Chancen der Verhütung, Früherkennung und optimierten Therapie chronischer Erkrankungen in der älteren Bevölkerung study; EVA, the Etude du Vieillissement Artériel study; HDL, high-density lipoprotein; ilSIRENTE, the Invecchiamento e Longevità nel Sirente study; InCHIANTI, the Invecchiare in Chianti, “Aging in the Chianti Area” study; LDL, low-density lipoprotein; MPP, the Malmö Preventive Project; NHANES, the National Health and Nutrition Examination Survey; PREVEND, the Prevention of REnal and Vascular End-stage Disease study; RR, risk ratio; SD, standard deviation; Se, selenium; SELENOP, selenoprotein P; WHAS, the Women's Health and Aging Study.

a mean ± SD or range of age.

b N_total_ was the number of participants analysed for all-cause mortality as long as it was assessed.

c Mean ± SD of Se status was shown for all studies, and the range of categories was shown for studies reported estimated effects across categories of Se status.

d RR (95 % CI) per SD increase of Se status.

e Age, sex, BMI or equivalent, and smoking.

f Calculated by deleting 8 % participants with missing data on covariates from 16,008 included participants.

g SD was calculated by the following equation: SD ≈ (P_80_ – P_20_)/1.68, assuming a normal distribution of Se status.

h SDs were calculated by the following equation: SD (study A) = Mean Se (study A) x SD (study B)/mean (study B), study A needed to have a similar mean Se concentration as study B and the same Se measurement method.

i Mean ± SD of plasma Se for females.

j The means were considered equivalent to the median under the assumption of a normal distribution of Se status.

k SDs were calculated by the following equation: SD ≈ (P_75_ – P_25_)/1.35, assuming a normal distribution of Se status.

l Additionally adjusted for serum cholesterol, systolic and diastolic blood pressure, week of blood collection, years of education, history of myocardial infarction, and history of stroke.

m Additionally adjusted for serum cholesterol, systolic and diastolic pressures, week of blood collection, years of education, and serum vitamins A and E.

n Additionally adjusted for cholesterol and drinking.

o Additionally adjusted for education, alcohol consumption, medication use, low cognitive function, diabetes, hypertension, dyslipidemia, history of cardiovascular disease.

p Additionally adjusted for black race, years of education, chronic obstructive pulmonary disease, peripheral arterial disease, angina, diabetes, and physical activity at baseline.

q Additionally adjusted for cognitive test score.

r Additionally adjusted for education, total energy intake, congestive heart failure, peripheral artery disease, stroke, Parkinson's disease and chronic obstructive pulmonary disease.

s Additionally adjusted for systolic blood pressure, number of prescribed drugs being taken, self-reported health score, physical activity score and receipt (or not) of certain state benefits (a potential index of relative poverty), baseline α1-antichymotrypsin, plasma creatinine, plasma total and HDL-cholesterol concentrations, plasma albumin concentration.

t Additionally adjusted for pack-years, spirits intake, dietary markers (salt and fat intake), chronic bronchitis and peak flow.

u Additionally adjusted for race–ethnicity, level of education, annual family income, serum cotinine concentration, alcohol consumption, fruit and vegetable intake, physical activity, serum total cholesterol concentration, hypertension status, diabetes status, history of heart attack, congestive heart failure, stroke or cancer, hormone use in women, and supplement use.

v Additionally adjusted for ischemic heart disease, diabetes, chronic obstructive pulmonary disease and ejection fraction <40 % according to echocardiography.

w Additionally adjusted for functional impairment, cancer, HDL-cholesterol concentration, interleukin-6 and CRP.

x Additionally adjusted for systolic blood pressure, use of antihypertensive medication, diabetes mellitus, LDL-cholesterol and HDL-cholesterol.

y Additionally adjusted for systolic blood pressure, total cholesterol, CRP, alcohol consumption, race, history of hypertension, history of cardiovascular disease, history of diabetes, estimated glomerular filtration rate, the use of lipid-lowering drugs and antiplatelet drugs.

z Additionally adjusted for drinking status, education level, physical activity, eGFR, hypertension, hyperlipidemia, diabetes at baseline, and future disease status, plasma metals, including antimony, copper, manganese, molybdenum, rubidium, thallium and vanadium. The model of CVD mortality further adjusted for family history of CVD.

{ Additionally adjusted for iron deficiency, anemia, CRP, cholesterol, glucose, and systolic blood pressure.

| Additionally adjusted for physical activity, alcohol consumption, history of cancer, cardiovascular disease, diabetes mellitus, dyslipidemia, inflammation (CRP ≥3 mg/L), and vitamin D status.

} Additionally adjusted for age, sex, BMI, race, education, diabetes, and hypertension.

∼ Additionally adjusted for age, gender, diastolic blood pressure, smoke, the family income‒to‒poverty ratio, hypertension, high total cholesterol, and stroke.

### 3.3 Risk of bias assessment

The quality of the included cohort studies, evaluated by the modified NOS, is shown in Appendix Table 6. Seventeen out of 20 studies scored 7 to 9 points and had a low risk of bias. Only three study scored 6 points and had a moderate risk of bias.

### 3.4 All-cause mortality

**Fig. 2:**
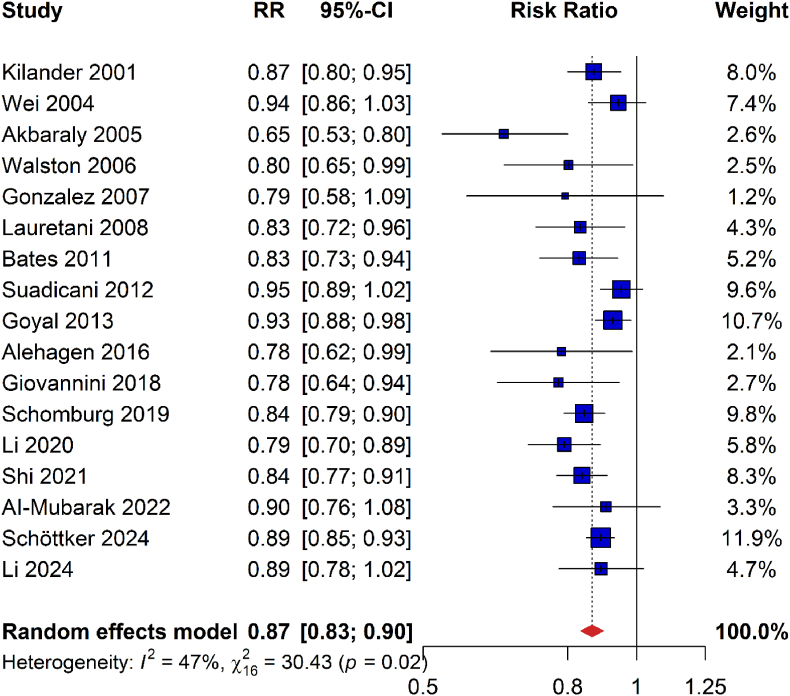
Meta-analysis of risk ratios for all-cause mortality per one standard deviation increase of plasma/serum selenium or selenoprotein P concentrations Abbreviations: 95 %-CI, 95 % confidence interval; RR, risk ratio.

Seventeen studies with 14,242 deaths among 61,576 participants reported sufficient data for analyzing the association of Se status with all-cause mortality [13,[15], [16], [17], [18], [19], [20],[39], [40], [41], [42], [43], [44], [45], [46], [47], [48]]. They consistently showed inverse associations, and random effects meta-analysis showed that all-cause mortality was statistically significantly reduced by 13 % per SD increment of Se/SELENOP concentration (RR [95 % CI], 0.87 [0.83–0.90]; Fig. 2).

### 3.5 Subgroup analyses for all-cause mortality

**Fig. 3:**
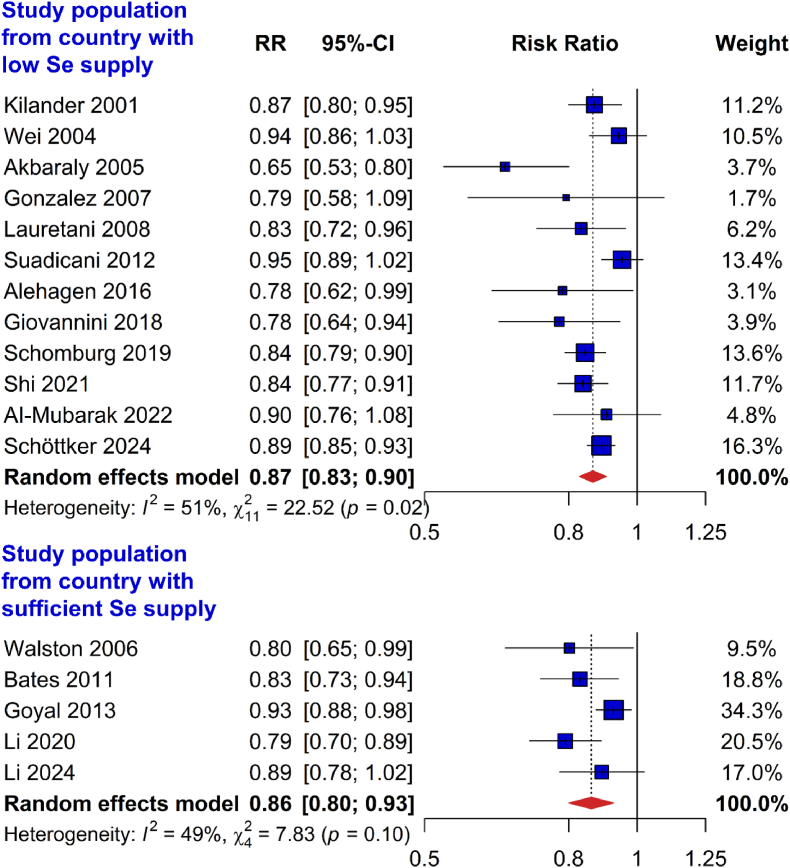
Subgroup meta-analyses by the background Se levels for the association of plasma/serum selenium or selenoprotein P concentrations (risk ratios per 1 standard deviation) with all-cause mortality Abbreviations: 95 %-CI, 95 % confidence interval; RR, risk ratio; Se, selenium.

**Fig. 4:**
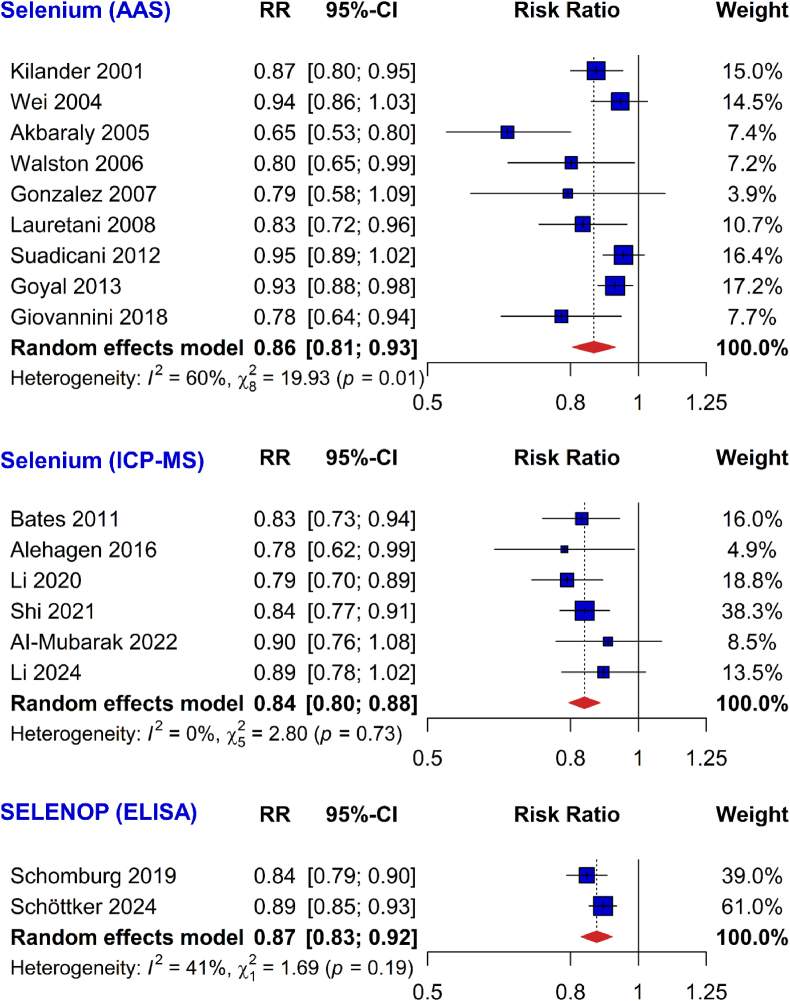
Subgroup meta-analyses by selenium measurement method for the association of selenium biomarkers (risk ratios per 1 standard deviation) with all-cause mortality Abbreviations: 95 %-CI, 95 % confidence interval; AAS, atomic absorption spectrometry; ELISA, enzyme-linked immunosorbent assay; RR, risk ratio; ICP-MS, inductively coupled plasma mass spectrometry; SELENOP, selenoprotein P.

In subgroup analysis by Se supply in the countries the study populations were recruited in, the inverse association of Se status with all-cause mortality was consistently observed in studies conducted in countries with low Se supply (0.87 [0.83–0.90]) and adequate Se supply (0.86 [0.80–0.93]; Fig. 3). Only 5 studies were included in the subgroup meta-analysis of countries with adequate Se supply and they were all either conducted in the UK (Bates 2011) or US (Walston 2006, Goyal 2013, Li 2020, and Li 2024). Similar results were also found in subgroup analyses by different measurement methods of Se status (plasma/serum Se measured using AAS: 0.86 [0.81–0.93]; plasma/serum Se measured using ICP-MS: 0.84 [0.80–0.88]; plasma/serum SELENOP using ELISA: 0.87 [0.83–0.92]; Fig. 4). Results were likewise consistent across recruitment years of the studies (before 1998: 0.87 [0.81–0.94]; 1998 or later: 0.85 [0.82–0.88]; Appendix Fig. 1), categories of study quality (NOS score <8: 0.85 [0.79–0.92]; NOS score ≥8: 0.87 [0.84–0.91]; Appendix Fig. 2), regions (Europe: 0.86 [0.82–0.90]; Asia: 0.89 [0.79–0.99]; North America: 0.87 [0.79–0.94]; Appendix Fig. 3), follow-up time (<10 years: 0.83 [0.80–0.86]; ≥10 years: 0.91 [0.88–0.94]; Appendix Fig. 4) and sample size (<3000 participants: 0.83 [0.78–0.88]; ≥3000 participants: 0.89 [0.86–0.93]; Appendix Fig. 5).

### 3.6 CVD mortality

**Fig. 5:**
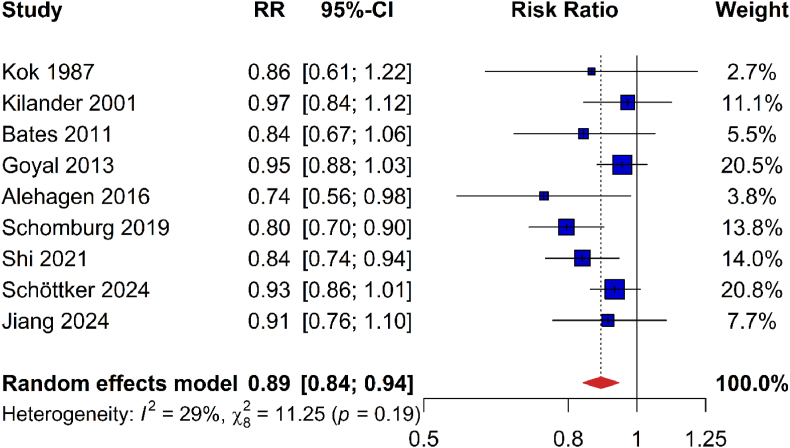
Meta-analysis of risk ratios for cardiovascular mortality per one standard deviation increase of plasma/serum selenium or selenoprotein concentrations Abbreviations: 95 %-CI, 95 % confidence interval; RR, risk ratio.

Nine studies with 4128 deaths due to CVD among 41,548 participants were included in the meta-analysis on the association of Se status with CVD mortality [13,15,16,20,35,39,44,46,49]. The pooled analysis demonstrated that CVD mortality was reduced by 11 % per SD increase of Se/SELENOP concentration (0.89 [0.84–0.94]; Fig. 5).

### 3.7 Cancer mortality

**Fig. 6:**
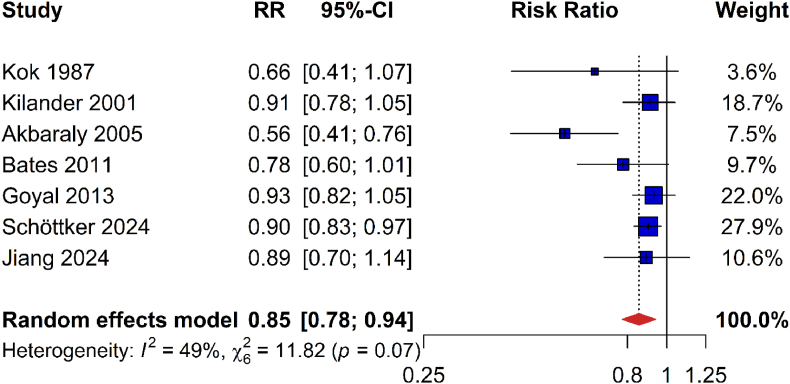
Meta-analysis of risk ratios for cancer mortality per one standard deviation increase of plasma/serum selenium or selenoprotein concentrations Abbreviations: 95 %-CI, 95 % confidence interval; RR, risk ratio.

Seven studies with 2156 deaths due to cancer among 31,823 participants were identified for the meta-analysis on cancer mortality [13,17,20,39,44,49,50]. The pooled risk estimate was statistically significant and showed 15 % decreased cancer mortality per SD Se/SELENOP increase (0.85 [0.78–0.94]; Fig. 6).

### 3.8 Sensitivity analyses

Sensitivity analyses excluding each single study showed that the inverse associations of Se status with all-cause mortality, CVD mortality and cancer mortality were not considerably influenced by any single study (data not shown).

### 3.9 Heterogeneity and publication bias

For the meta-analysis of all-cause mortality, heterogeneity was moderate and statistically significant (I^2^, 47 %; P = 0.02; Fig. 2). The funnel plot of the study results was asymmetrical with more published larger studies reporting relatively weak risk estimates and more published small studies reporting relatively strong risk estimates (Appendix Fig. 6). Not surprisingly, the Egger's test for publication bias was statistically significant (P = 0.015). Imputing the potentially unpublished 6 studies with the trim and fill method resulted in a slightly weaker but still statistically significant risk estimate (0.89 [0.85–0.92] instead of 0.87 [0.83–0.90]; see Appendix Fig. 7 for the funnel plot with filled studies).

In the meta-analysis of CVD mortality, the heterogeneity was not important and not statistically significant (I^2^, 29 %; P = 0.19; Fig. 5). Furthermore, there was no indication of publication bias (see Appendix Fig. 8 for funnel plot; Egger's test, P = 0.178). The meta-analysis for cancer mortality showed moderate but not statistically significant heterogeneity (I^2^, 49 %; P = 0.07; Fig. 6). Furthermore, there was no evidence for publication bias (see Appendix Fig. 9 for funnel plot; Egger's test, P = 0.07).

## 4 Discussion

This systematic review and meta-analysis, including 20 studies with overall 61,576 study participants, showed that Se/SELENOP concentration is inversely associated with all-cause mortality, CVD mortality and cancer mortality. Although statistically significant heterogeneity and publication bias were observed for all-cause mortality, the results proved to be robust in several additional analyses. For example, results did not differ substantially by Se supply in the countries, Se measurement methods, time of baseline recruitment, study quality, study region, follow-up length and sample size. Almost all studies had a low risk of bias and were adjusted for a key set of potential confounders.

### 4.1 Comparison with previous systematic reviews of cohort studies on mortality outcomes

The meta-analyses by Jayedi et al. and Xiang et al., summarizing 7 and 8 studies with less than 24,000 study participants, have both reported reduced all-cause mortality comparing the highest category of circulating Se with the lowest (Jayedi et al., 0.62 [0.45–0.79]; Xiang et al., 0.74 [0.63–0.85]) [21,23]. However, these risk estimates were difficult to interpret, because the cut-off values for the highest and lowest categories of circulating Se varied strongly across the included studies. These two meta-analyses also pooled 6 and 5 studies for continuous risk estimates, and reported an 11 % (0.89 [0.84–0.95]) reduced all-cause mortality for each 0.20 μmol/L increase of circulating Se [21], and a 20 % reduced all-cause mortality per SD increment (0.80 [0.70–0.92]), respectively [23]. However, the number of included studies had been low at the time of their analysis. Our analysis which included more recently published results of large cohorts obtained a more precise risk estimate on the inverse association of Se/SELENOP concentration per 1 SD with all-cause mortality (0.86 [0.82–0.89]) by pooling 17 studies with a total of 61,576 study participants.

Regarding CVD mortality, the meta-analysis by Xiang et al. summarised 4 observational studies with 17,494 study participants and reported a 26 % lower CVD mortality for the highest versus the lowest category of circulating Se (0.74 [0.62–0.88]) [23]. Another meta-analysis by Kuria et al. included 7 studies and reported a similar risk estimate when comparing the highest and lowest Se categories (0.70 [0.60–0.80]), although this meta-analysis was not restricted to population based observational studies [22]. Kuria et al. conducted an additional meta-analysis with 7 studies for a 10 μg/L increase in blood Se concentration but observed no statistically significant association with CVD mortality (0.93 [0.83–1.05]). Our more up-to-date meta-analysis summarised 9 cohort studies with 41,548 participants, and showed a significant reduction of CVD mortality by 11 % per SD increase of Se/SELENOP concentration (0.89 [0.83–0.94]).

To the best of our knowledge, no other meta-analysis of population-based observational studies on the association of blood Se status with cancer mortality has been conducted before. A previous systematic review and meta-analysis pooled 7 cohort studies with varying Se exposure definitions including dietary Se intake [51]. Although its results are not directly comparable to ours, it is of interest to note that they also detected a statistically significant inverse association of Se exposure with cancer mortality (0.76 [0.59–0.97]) for comparison of the highest with the lowest Se category.

### 4.2 Comparison with previous systematic reviews of randomized controlled trials (RCTs) on mortality outcomes

Meta-analysis of RCTs comparing Se supplementation with placebo did not observe reductions in all-cause mortality, CVD mortality or cancer mortality [52,53]. The absence of significant findings in these meta-analyses is probably due to the inclusion of the very large Selenium and Vitamin E Cancer Prevention Trial (SELECT), which dominated the results in the meta-analyses [54]. The SELECT trial was conducted in the United States, Canada and Puerto Rico, which are regions with a high Se concentration in soil. Se deficiency was not an inclusion criterion of the trial and the baseline serum Se concentration of SELECT study participants was much higher (median, 135 μg/L) than the cut-off frequently used to define suboptimal Se concentration (100 μg/L) [55]. Thus, the population of the SELECT study could not profit from Se supplementation because there was no need for higher serum Se concentration in the great majority of the study participants.

We would like to highlight one particular RCT from Denmark, which is a European country with low Se supply, which excludes the limitation of the SELECT trial [56]. The Denmark PRECISE study observed a hazard ratio (HR) (95 % CI) for all-cause mortality comparing 300 μg Se/d to placebo of 1.62 (0.66, 3.96) after 5 years of treatment and 1.59 (1.02, 2.46) over the entire follow-up period of 10 years. The surprising direction of the effect should be treated with caution because the Denmark PRECISE study was quite small (n = 491). It observed 31 deaths during the 5-year period with Se supplementation and the HR for all-cause mortality was not statistically significant and had a very wide confidence interval. The analysis of the 10-year follow-up has a higher statistical power (n = 158) but half of the follow-up time, in which 80 % of the cases occurred, is observational without treatment. To verify if there is an increased mortality by Se supplementation, a new trial with 10-year Se supplementation would be needed.

### 4.3 The roles of selenoproteins in human health

Se exists naturally in water and soil, and thereby food [57]. Dietary Se is absorbed from the duodenum and cecum [58] and delivered to the liver, where SELENOP is synthesized. SELENOP transports Se to the targeted tissues, such as the brain, thyroid gland, kidney, for the synthesis of other selenoproteins (GPXs, TXNRDs, and iodothyronine deiodinases (DIO)), which are involved in several metabolic and functional pathways [6,[59], [60], [61]].

**Fig. 7:**
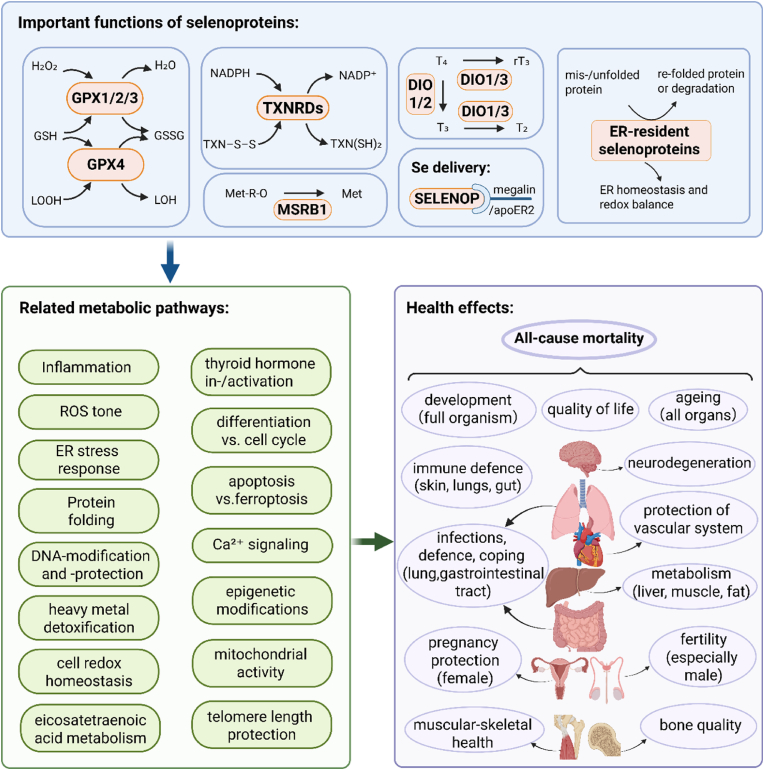
Functions, metabolic pathways and health effects of selenoproteins ^a^ Abbreviations: GPXs, glutathione peroxidases; H_2_O_2_, hydrogen peroxide, LOOH, lipid hydroperoxides; GSH, reduced glutathione; GSSG, oxidized glutathione; NADPH, reduced form of nicotinamide adenine dinucleotide phosphate; NADP^+^, oxidized form of nicotinamide adenine dinucleotide phosphate; TXNRDs, thioredoxin reductases; TXN–S–S, oxidized thioredoxin; TXN(SH)_2_, reduced form of thioredoxin; MSRB1, methionine sulfoxide reductase B1; Met-R-O, methionine-R-sulfoxide; Met, methionine; DIOs, iodothyronine deiodinases; T_4_, thyroxine; T_3_, triiodothyronine; rT_3_, reverse triiodothyronine; T_2_, diiodothyronine; ER, endoplasmic reticulum; SELENOP, selenoprotein P; ROS, reactive oxygen species. ^a^ Created in BioRender. Cui, Z. (2025) https://BioRender.com/psa1qx3.

A mechanistic scheme of the roles of selenoproteins in human health is shown in Fig. 7. The role of some selenoproteins is still elusive, but several members are well characterized in terms of activity and specific functions.

• Glutathione peroxidases (GPXs) reduce hydrogen peroxide (H_2_O_2_) and lipid hydroperoxides (LOOH) [62];
• Thioredoxin reductases (TXNRDs) catalyze the reduction of e.g. oxidized thioredoxin (TXN–S–S) to its active form TXN(SH)_2_ [62];
• Methionine sulfoxide reductase B1 (MSRB1) catalyzes the reduction of methionine-R-sulfoxide (Met-R-O) back to methionine (Met) [62];
• Iodothyronine deiodinases (DIOs) catalyze the activation of thyroxine (T_4_) to triiodothyronine (T_3_), or the conversion of T_4_ to inactive rT_3_ or of active T3 to inactive diiodothyronine (T_2_) [61,63];
• SELENOP ensures Se transport and hierarchical supply of essential organs [6,64];
• Selenoproteins of the endoplasmic reticulum (ER) contribute to protein glycosylation, maturation and quality control of intraluminal, membrane-bound or secreted proteins [65].

Several important metabolic pathways and biochemical processes are affected by these diverse activities, including inflammation [61,66], reactive oxygen species (ROS) tone [60], ER stress response [65], protein folding [65], DNA modifications [61], heavy metal detoxification [66], the redox homeostasis of cells [66], eicosatetraenoic acid metabolism [66], thyroid hormone activity [67], cell differentiation or proliferation [66], the extent of cell loss through apoptosis or ferroptosis [68,69], Ca signalling [66], epigenetic modifications [69], mitochondrial activity [70] and the protection of telomere length [71].

Accordingly, Se deficiency impairs the optimal expression of certain selenoproteins in the various tissues, as the organism must prioritise its distribution to the sites with the highest relevance, including the central nervous system, the immune system and the endocrine system (triage concept). Certain health effects and increased risks result from Se deficiency, either for specific tissues or for the whole organism, including but not limited to: developmental processes [72,73], quality of life [73], ageing [74], neurodegeneration [75], cardiovascular diseases and events [66], immune responses in the skin, lungs and gut [76,77], cancer risks at various sites [66], autoimmune diseases, especially of the thyroid gland [78], dysglycaemia due to impaired metabolism of micro- and macronutrients [6], degenerative defects of the vascular [60], muscular and skeletal systems [79], as well as impaired fertility, especially in terms of sperm quality [78], and health risks during pregnancy for mother and foetus [6], postpartum and developmental problems [80,81], as well as overcoming bacterial or viral infections, as observed during the recent COVID pandemic [82]. It is noteworthy that many of the few published intervention studies reported an improvement in quality of life after correction of Se deficiency [83,84], indicating the importance of selenoproteins for the regular function of several important biochemical, neurological and endocrine pathways. Thus, given the essential role of selenoproteins for human health, the observed association of low Se blood concentrations with all-cause mortality is supported by biological evidence.

### 4.4 Se and cardiovascular disease

The main cause of CVD is atherosclerosis [85]. Oxidative stress and endothelial dysfunction are implicated in the atherosclerosis development, which is a chronic inflammatory status with increased pro-inflammatory cytokines [86,87]. Selenoproteins may prevent atherosclerosis by improving both vascular function and the pro-inflammatory state. Selenoproteins regulate vasoreactivity by reducing ROS and preserving bioavailable nitric oxide. It was shown that GPX1 knockout mice had increased ROS formation and dysregulation of endothelial nitric oxide (NO) synthase [88]. The supplementation of sodium selenite can increase GPX1 activity in humans with coronary artery disease (CAD) and GPX1 activity was shown to be inversely associated with cardiovascular events in a large prospective study with CAD patients [89,90].

Furthermore, improved total anti-oxidant status, reduced oxidative stress and increased NO bioavailability were observed in mice with endothelial cell-specific overexpression of TXNRD2 [91]. Besides, as the TXNRDs possess a broad substrate specificity, they can contribute to the reduction of non-disulfides and the activation of antioxidants, such as ascorbic acid and α-Lipoic acid [92,93]. Regarding inflammation, lack of GPX1 in mice augmented the nuclear factor-κappaB activity and prolonged the activation of the mitogen-activated protein kinase pathway [94]. A similar result was also found in mice with a knockdown of selenoprotein S [95]. In addition, GPX4 was reported to reduce phospholipid hydroperoxide and regulate the oxidation of lipid [96].

With respect to CVD incidence in humans, a meta-analysis of 3 cohort studies showed that there was a 20 % decreased CVD incidence risk for participants in physiologically high Se body status (0.80 [0.70–0.92]), though no statistical association was observed when pooling 13 case-control studies [22]. A more recent publication of the NHANES, including 23,448 participants, also showed that higher blood Se was associated with a lower risk of CVD (0.65 [0.57–0.74]) [48]. However, a meta-analysis of 2 RCTs showed no statistically significant effect of Se supplementation on CVD incidence [52].

### 4.5 Se and cancer

With respect to cancer, the anti-oxidative actions of selenoproteins stated above for prevention of CVD may also prevent DNA damage [6,97]. Moreover, selenomethionine may stimulate DNA damage repair [98,99]. Interestingly, variations in genes coding for the biosynthesis of selenoproteins, such as rs11111979 for TXNRD1, were found to modify the risk of colorectal cancer development [100].

A meta-analysis and systematic review showed that higher blood/toenail Se was associated with a lower risk of total cancer, and a similar trend was observed between Se and lung, prostate, breast, oesophageal and gastric cancer [101]. An umbrella review of meta-analyses also showed that Se intake was associated with a reduced risk of digestive system cancers [78]. A meta-analysis of 4 RCTs showed a reduced risk of liver cancer incidence for Se supplementation (0.52 [0.35–0.79]) [51]. Of note, some RCTs raised concerns about higher incidences of prostate and skin cancer by taking Se supplements [51,102].

### 4.6 Se and other diseases

In terms of other diseases, a meta-analysis and systematic review showed that the measured blood Se status and the Se intake are inversely associated with the risk of hepatitis and cirrhosis [103]. Furthermore, Se supplementation was shown to improve polycystic ovary syndrome and autoimmune thyroid disorders [78].

### 4.7 Strengths and limitations

This study has several strengths. Firstly, we were able to pool studies with results reported either by categorical or continuous Se biomarker variables in the same meta-analysis, which resulted in more precise pooled risk estimates. Secondly, we included studies using either serum/plasma Se or SELENOP measurements in the same meta-analysis, which increased the sample size a lot because the studies using SELENOP measurements were very large. Thirdly, we performed several subgroup analyses (e.g., by Se supply in the countries, Se measurement methods, time of baseline recruitment, study quality, study region, follow-up length and sample size) to explore potential sources of heterogeneity in the association of Se status with all-cause mortality. Unfortunately, these subgroup analyses could not be carried out for CVD and cancer mortality due to the limited number of studies for these outcomes. Fourthly, we present the first systematic review with a sufficient number of studies for a meta-analysis on cancer mortality.

Some limitations should be considered when interpreting the results of the meta-analyses. We performed the generalized least squares for linear trend estimation under the assumption that there was a linear dose-response relationship between Se status with the outcomes of interest. A previous meta-analysis tested for non-linearity in the association of circulating Se with all-cause mortality and did not observe a deviation from linearity (*P* for non-linearity = 0.40) [21]. In contrast, large studies from Germany, the Netherlands and the US-American NHANES observed an L-shaped association of SELENOP/Se with all-cause mortality, which is biologically more plausible [13,14,18]. There is only one older study from the NHANES, which observed a U-shaped association, and this was conducted among US-American citizens with quite high Se plasma concentrations [104]. However, in the relevant range of SELENOP/Se concentration below the median, this L-shaped association is close to being linear. Thus, although the association of Se biomarker concentration with mortality outcomes might not be perfectly linear, the association is close enough to linearity to allow the usage of linear trend estimation. This is a compromise that gives valid estimates for the general population. Effect estimates for subjects with low Se status are likely stronger and effect estimate for subject with high Se status are likely weaker than those reported for the general population. A dose-response meta-analysis or pooling risk estimates associated with 1-unit increase in circulating Se/SELENOP concentrations was not possible because the Se measurement methods were not standardized across studies and absolute Se values were not comparable. Dose-response relationships can be obtained from the individual studies cited above [13,14,18].

In addition, two studies published prior to the year 2000 reported RRs while all others used HRs. HRs and RRs provide similar results when the risk of the event of interest is low and the follow-up time is relatively short. This was the case for the two older studies, which analysed 84 and 69 deaths in 6–9 years of follow-up [35,50]. Thus, we think it was feasible to pool all studies. We also checked that excluding the two older studies did not alter the results substantially (data not shown).

Furthermore, indications of a publication bias were observed in the meta-analysis on all-cause mortality. However, the pooled risk estimate was only slightly diminished, and was still statistically significant after including filled studies using the trim and fill method. In addition, there was moderate heterogeneity among the included studies, which could not be explained by the conducted subgroup analyses. The heterogeneity might result from a sex difference because twice as strong associations of Se status with mortality have been found among men than among women [13] and some studies were conducted among men or women only [39,41,45]. Additionally, residual confounding from unconsidered potential confounders should be noted as a limitation, which cannot be excluded in observational studies. Finally, as the included studies have mainly been conducted in middle-aged and older adults from the general population, the findings cannot be generalized to young adults and frail elderly.

### 4.8 Areas requiring further research

To start with, the potential sex difference in the association of Se status and all-cause mortality should be further explored. The ESTHER study from Germany, the PREVEND study from the Netherlands and the NHANES study (1999–2006) from the United States have reported a stronger association of Se status with all-cause mortality in men than in women [13,18,19], but no sex differences were reported in the NHANES III study [20]. We did not conduct a subgroup meta-analysis by sex because studies reporting risk estimates separately for men and women were too few (only the 4 studies stated above reported these data).

In addition, potential age differences concerning the association of Se status and all-cause mortality should also be explored. Only two studies reported results for different age groups [13,20]. One is the German ESTHER study, conducted among participants aged 50–75 years, which did not observe large differences between the age groups 50–64 and 65–75 years [13]. The other is the NHANES III study, which observed comparable statistically significant associations of circulating Se with all-cause mortality among participants aged 40–59 years and ≥60 years, but did not find an association in young adults aged 20–39 years [20]. We abstained from conducting subgroup meta-analysis by age because studies reporting risk estimates in different age groups were too few (only the two stated above) and most studies had populations with a narrow age range with mean ages higher than 60 years.

Regarding RCTs, the gold standard study design, we call for new, well-designed RCTs with long-term treatment to evaluate if and to what extent Se supplementation could reduce mortality among study populations with low plasma Se < 100 μg/L as an inclusion criterion. However, the dose should be selected with caution because some RCTs reported potential adverse effects of Se supplementation, such as type 2 diabetes [105], prostate cancer [51], skin cancer [102], and mortality [56].

### 4.9 Public health implications

The alleviation of Se deficiency may have profound public health implications. It is reported that 500 million to 1 billion of the global population are exposed to Se deficiency, mainly because of inadequate dietary intake [106]. The dietary reference intakes of Se are different due to various distributions of global Se content. The European Food Safety Authority has set the adequate intake of Se at 70 μg/day for adults [107]. The recommended dietary allowance of Se is 55 μg/day for adults in the United States [108]. Se deficiency could be overcome by dietary intake of food rich in Se. Meat, seafood, cereals and grains are foods with high Se content if they come from regions with Se-rich soil [109]. Therefore, people who live in regions with low soil Se concentration or who do not eat enough Se-rich foods may need to take Se supplements.

## 5 Conclusion

This systematic review and meta-analysis observed that Se status is inversely associated with all-cause mortality, CVD mortality and cancer mortality. Although statistically significant between-study heterogeneity in the meta-analyses was observed, the inverse association with all-cause mortality was robust across the Se supply in study populations from different countries, different study regions, Se measurement methods, study recruitment years, follow-up lengths, study sizes, and study qualities. Although there were indications of publication bias in the meta-analysis of all-cause mortality, there was still a statistically significant risk estimate after imputing the potentially unpublished studies with the trim and fill method. Well-designed, large trials on Se supplementation, which should be conducted among participants with low Se status, are still needed. If such trials confirm causality of the observed inverse relationships of Se with all-cause and cause-specific mortality reported by this systematic review and meta-analysis of 20 observational studies, and provide evidence for beneficial effects of Se supplementation, such supplementation could be a highly cost-effective and safe intervention to prolong the healthy life-span among older adults with Se deficiency.

## CRediT authorship contribution statement

**Zhixin Cui:** Writing – original draft, Project administration, Methodology, Investigation, Formal analysis, Data curation, Conceptualization. **Ruijie Xie:** Investigation, Data curation. **Xiaoting Lu:** Investigation, Data curation. **Lutz Schomburg:** Writing – review & editing. **Hermann Brenner:** Writing – review & editing. **Ben Schöttker:** Writing – review & editing, Supervision, Methodology, Data curation, Conceptualization.

## Disclosure statement

L.S. holds shares in selenOmed GmbH, Berlin, a company involved in Se status assessment. All other authors have no conflicts of interest.

## Data Availability

Data will be made available on request.
