# Supplementary material for: Associations of selenium status with all-cause and cause-specific mortality: a systematic review and meta-analysis of cohort studies
Source: Redox Biol. 2025 Jul 10;85:103755. doi: 10.1016/j.redox.2025.103755 (PMC12302194; doi:10.1016/j.redox.2025.103755)
Supplement: Multimedia component 1 [file mmc1.docx]

**Appendix to the Article:**

**“Associations of selenium status with all-cause mortality and cause-specific mortality: a systematic review and meta-analysis of observational studies”**

# **Table of Contents**

[**Appendix Table 1.** MOOSE Checklist 2](#_Toc195612160)

[**Appendix Table 2.** Search strategy for MEDLINE via PubMed on 11.03.2025 5](#_Toc195612161)

[**Appendix Table 3.** Search strategy for ISI Web of Knowledge on 11.03.2025 6](#_Toc195612162)

[**Appendix Table 4.** Modified Newcastle-Ottawa Quality Assessment Scale for Cohort Studies 7](#_Toc195612164)

[**Appendix Table 5.** Reasons and references of excluded studies 8](#_Toc195612163)

[**Appendix Table 6.** Quality of studies evaluated for cohort studies according to a modified Newcastle-Ottawa-Scale 12](#_Toc195612165)

[**Appendix Figure 1.** Subgroup meta-analyses by recruitment years of the studies for the association of plasma/serum selenium or selenoprotein P concentration (risk ratios per 1 standard deviation) with all-cause mortality 13](#_Toc195612166)

[**Appendix Figure 2.** Subgroup meta-analyses by the modified Newcastle-Ottawa scale scores for the association of plasma/serum selenium or selenoprotein P concentration (risk ratios per 1 standard deviation) with all-cause mortality 14](#_Toc195612167)

[**Appendix Figure 3.** Subgroup meta-analyses by region for the association of plasma/serum selenium or selenoprotein P concentrations (risk ratios per 1 standard deviation) with all-cause mortality 15](#_Toc195612168)

[**Appendix Figure 4.** Subgroup meta-analyses by follow-up time for the association of plasma/serum selenium or selenoprotein P concentrations (risk ratios per 1 standard deviation) with all-cause mortality 16](#_Toc195612169)

[**Appendix Figure 5.** Subgroup meta-analyses by sample size for the association of plasma/ serum selenium or selenoprotein P concentrations (risk ratios per 1 standard deviation) with all-cause mortality 17](#_Toc195612170)

[**Appendix Figure 6.** Funnel plot for the meta-analysis on the association of selenium status with all-cause mortality 18](#_Toc195612174)

[**Appendix Figure 7.** Funnel plot for the meta-analysis on the association of selenium status with all-cause mortality, including filled studies using trim and fill method 19](#_Toc195612175)

[**Appendix Figure 8.** Funnel plot for the meta-analysis on the association of selenium status with cardiovascular mortality 20](#_Toc195612176)

[**Appendix Figure 9.** Funnel plot for the meta-analysis on the association of selenium status with cancer mortality 21](#_Toc195612177)

# **Appendix Table 1.** MOOSE Checklist

| **Reporting Criteria** | **Position of Relevant Report** |
| --- | --- |
| **Reporting of Background** |  |
| 1. Problem definition | Introduction, paragraph 3 |
| 1. Hypothesis statement | Introduction, paragraph 4 |
| 1. Description of study outcome(s) | Methods, chapter 2.2 Eligibility criteria and data extraction, paragraph 2 |
| 1. Type of exposure or intervention used | Methods, chapter 2.2 Eligibility criteria and data extraction, paragraph 2 |
| 1. Type of study designs used | Methods, chapter 2.2 Eligibility criteria and data extraction, paragraph 2 |
| 1. Study population | Methods, chapter 2.2 Eligibility criteria and data extraction, paragraph 2 |
| **Reporting of Search Strategy** |  |
| 1. Qualifications of searchers (e.g. librarians and investigators) | Methods, chapter 2.2 Eligibility criteria and data extraction, paragraph 3 |
| 1. Search strategy, including time period included in the synthesis and keywords | Methods, chapter 2.1 Search strategy |
| 1. Effort to include all available studies, including contact with authors | Methods, chapter 2.2 Eligibility criteria and data extraction, paragraph 2, and chapter 2.4 Data synthesis and statistical analysis, paragraph 1-11 |
| 1. Databases and registries searched | Methods, chapter 2.1 Search strategy |
| 1. Search software used, name and version, including special features used (e.g. explosion) | Methods, chapter 2.1 Search strategy |
| 1. Use of hand searching (e.g. reference lists of obtained articles) | Methods, chapter 2.2 Eligibility criteria and data extraction, paragraph 2 |
| 1. List of citations located and those excluded, including justification | Appendix Table 5. Reasons and references of excluded studies |
| 1. Method of addressing articles published in languages other than English | Methods, chapter 2.1 Search strategy |
| 1. Method of handling abstracts and unpublished studies | No abstracts indicating unpublished studies were identified. |
| 1. Description of any contact with authors | Methods, chapter 2.2 Eligibility criteria and data extraction, paragraph 2 |
| **Reporting of Methods** |  |
| 1. Description of relevance or appropriateness of studies assembled for assessing the hypothesis to be tested | Methods, chapter 2.1 Search strategy to 2.4 Data synthesis and statistical analysis |
| 1. Rationale for the selection and coding of data (e.g. sound clinical principles or convenience) | Methods, chapter 2.1 Search strategy to 2.4 Data synthesis and statistical analysis |
| 1. Documentation of how data were classified and coded (e.g. multiple raters, blinding, and interrater reliability) | Methods, chapter 2.1 Search strategy and 2.4 Data synthesis and statistical analysis |
| 1. Assessment of confounding (e.g., comparability of cases and controls in studies where appropriate) | Methods, chapter 2.4 Data synthesis and statistical analysis, paragraph 12 |
| 1. Assessment of study quality, including blinding of quality assessors; stratification or regression on possible predictors of study results | Methods, chapter 2.3 Assessment of study quality |
| 1. Assessment of heterogeneity | Methods, chapter 2.4 Data synthesis and statistical analysis, paragraph 12 |
| 1. Description of statistical methods (e.g. complete description of fixed or random effects models, justification of whether the chosen models account for predictors of study results, dose-response models, or cumulative meta-analysis) in sufficient detail to be replicated | Methods, chapter 2.4 Data synthesis and statistical analysis, paragraph 12 |
| 1. Provision of appropriate tables and graphics | Table1; Figure 1 to 7; Appendix Table 1 to 6; Appendix Figure 1 to 9 |
| **Reporting of Result** |  |
| 1. Table giving descriptive information for each study included | Table1 |
| 1. Results of sensitivity testing (e.g. subgroup analysis) | Results, chapter 3.5 Subgroup analyses for all-cause mortality; Figure 3 and 4; Appendix Figure 1-5; chapter 3.8 Sensitivity analyses |
| 1. Indication of statistical uncertainty of findings | Results, chapter 3.8 Heterogeneity and publication bias; Figure 2, 5 and 6; Appendix Figure 6-9 |
| **Reporting of Discussion** |  |
| 1. Quantitative assessment of bias (e.g., publication bias) | Discussion, chapter 4.3 Strengths and limitations, paragraph 4 |
| 1. Justification for exclusion (e.g. exclusion of non–English-language citations) | Discussion, chapter 4.3 Strengths and limitations, paragraph 4 |
| 1. Assessment of quality of included studies | Discussion, paragraph 1 |
| **Reporting of Conclusions** |  |
| 1. Consideration of alternative explanations for observed results | Discussion, chapter 4. 6 Conclusion |
| 1. Generalization of the conclusions (i.e. appropriate for the data presented and within the domain of the literature review) | Discussion, chapter 4. 6 Conclusion |
| 1. Guidelines for future research | Discussion, chapter 4. 6 Conclusion |
| 1. Disclosure of funding source | Acknowledgements |

# **Appendix Table 2.** Search strategy for MEDLINE via PubMed on 11.03.2025

| **Search No.** | **Search query** | **Hits** |
| --- | --- | --- |
| 1 | (mortality) OR (death[Title/Abstract]) | 2,363,852 |
| 2 | (((selen*) OR (selenium[MeSH Terms])) OR ("plasma Se")) OR ("serum Se") | 63,793 |
| 3 | ((((((randomized controlled trial[Publication Type]) OR (randomized controlled trial*[Title])) OR (review[Publication Type])) OR (editorial[Publication Type])) OR (letter[Publication Type])) OR (animal[Filter])) OR (cell*[Title]) | 14,120,002 |
| 4 | #1 AND #2 NOT #3 | 1,040 |

# **Appendix Table 3.** Search strategy for ISI Web of Knowledge on 11.03.2025

| **Search No.** | **Search query** | **Hits** |
| --- | --- | --- |
| 1 | (TS=(mortality)) OR TS=(death) | 4,439,214 |
| 2 | TS= ("selen*" OR "plasma Se" OR "serum Se") | 269,960 |
| 3 | #1 AND #2 | 8,299 |
| 4 | #1 AND #2 and Humans (MeSH Heading) | 3,222 |
| 5 | #1 AND #2 and Review Article or Meeting or Patent or Clinical Trial or Book or Editorial Material or Letter or Case Report or News (Document Types) | 2,529 |
| 6 | #1 AND #2 and Agriculture or Zoology or Veterinary Sciences or Plant Sciences (Research Areas) | 2,119 |
| 7 | #1 AND #2 and Animals or Apoptosis or Mice or Rats or Cell Survival or Cell Death or Cell Proliferation or Cells Cultured (MeSH Headings) | 2,829 |
| 8 | (((#4) NOT #5) NOT #6) NOT #7 | 1,061 |

# **Appendix Table 4.** Modified Newcastle-Ottawa Quality Assessment Scale for Cohort Studies

| **SELECTION** |
| --- |
| 1. Representativeness of the Exposed Cohort  a) truly representative of the average selenium exposed people in the community (1 point)  b) somewhat representative of the average selenium exposed people in the community (1 point)  c) selected group of users (e.g. nurses, volunteers)  d) no description of the derivation of the cohort |
| 2. Selection of the Non-Exposed Cohort  a) drawn from the same community as the expected cohort (1 point)  b) drawn from a different source  c) no description of the derivation of the non-exposed cohort |
| 3. Ascertainment of Exposure  a) secure record (1 point)  b) structured interview  c) written self report  d) no description |
| 4. Demonstration That Outcome of Interest Was Not Present at Start of Study  a) yes, a statement of no history of disease or incident (1 point)  b) no |
| **COMPARABILITY** |
| 1. Comparability of Cohorts on the Basis of the Design or Analysis  a) study controls for age, sex, body mass index or equivalent, and smoking (1 point)  b) study controls for any additional factor other than the most important factor (the second point) |
| **OUTCOME** |
| 1. Assessment of Outcome  a) independent or blind assessment (1 point)  b) record linkage (e.g. identified through ICD codes on database records) (1 point)  c) self-report  d) no description |
| 2. Was Follow-Up Long Enough for Outcomes to Occur  a) yes, at least 4-year follow-up (1 point)  b) no |
| 3. Adequacy of Follow Up of Cohorts  a) yes, at least 80% follow up (1 point)  b) < 80% follow up or not mentioned |

# **Appendix Table 5.** Reasons and references of excluded studies

| **(A) No observational study design (3 studies)** |
| --- |
| 1. Kok FJ, De Bruijn AM, Hofman A, Valkenburg HA. Selenium status and chronic disease mortality: Dutch epidemiological findings. Int J Epidemiol. 1987 Jun; 16(2):329-32. |
| 2. He X, Sun H, Zhao Y, Fu X, Wang M, Liu M, Su Y, Hu F, Qin P, Zhang M, Hu D. Association of environmental metallic and metalloid contaminants with cardiovascular and all-cause mortality: An umbrella review of systematic reviews and meta-analyses. Eur J Prev Cardiol. 2025 Mar 3:zwaf118. |
| 3. Alexander J, Aaseth JO, Schomburg L, Chillon TS, Larsson A, Alehagen U. Circulating Glutathione Peroxidase-3 in Elderly-Association with Renal Function, Cardiovascular Mortality, and Impact of Selenium and Coenzyme Q10 Supplementation. Antioxidants (Basel). 2024 Dec 19;13(12):1566. |
| **(B) No selenium status measurement (1 study)** |
| 1. Nakadaira H, Endoh K, Yamamoto M, Katoh K. Distribution of selenium and molybdenum and cancer mortality in Niigata, Japan. Arch Environ Health. 1995 Sep-Oct; 50(5):374-80. |
| **(C) No relative risk effect suitable for meta-analysis (14 studies)** |
| 1. Chen J, Geissler C, Parpia B, Li J, Campbell TC. Antioxidant status and cancer mortality in China. Int J Epidemiol. 1992 Aug; 21(4):625-35. |
| 2. Criqui MH, Bangdiwala S, Goodman DS, Blaner WS, Morris JS, Kritchevsky S, Lippel K, Mebane I, Tyroler HA. Selenium, retinol, retinol-binding protein, and uric acid. Associations with cancer mortality in a population-based prospective case-control study. Ann Epidemiol. 1991 Aug; 1(5):385-93. |
| 3. Demircan K, Bengtsson Y, Chillon TS, Vallon-Christersson J, Sun Q, Larsson C, Malmberg M, Saal LH, Rydén L, Borg Å, Manjer J, Schomburg L. Matched analysis of circulating selenium with the breast cancer selenotranscriptome: a multicentre prospective study. J Transl Med. 2023 Sep 23; 21(1):658. |
| 4. Detopoulou P, Letsiou S, Nomikos T, Karagiannis A, Pergantis SA, Pitsavos C, Panagiotakos DB, Antonopoulou S. Selenium, Selenoproteins and 10-year Cardiovascular Risk: Results from the ATTICA Study. Curr Vasc Pharmacol. 2023; 21(5):346-355. |
| 5. Guo WD, Li JY, Blot WJ, Hsing AW, Chen JS, Fraumeni JF Jr. Correlations of dietary intake and blood nutrient levels with esophageal cancer mortality in China. Nutr Cancer. 1990; 13(3):121-7. |
| 6. Guo W, Zheng W, Li JY, Chen JS, Blot WJ. Correlations of colon cancer mortality with dietary factors, serum markers, and schistosomiasis in China. Nutr Cancer. 1993; 20(1):13-20. |
| 7. Guo WD, Hsing AW, Li JY, Chen JS, Chow WH, Blot WJ. Correlation of cervical cancer mortality with reproductive and dietary factors, and serum markers in China. Int J Epidemiol. 1994 Dec; 23(6):1127-32. |
| 8. Guo WD, Chow WH, Zheng W, Li JY, Blot WJ. Diet, serum markers and breast cancer mortality in China. Jpn J Cancer Res. 1994 Jun; 85(6):572-7. |
| 9. Kneller RW, Guo WD, Hsing AW, Chen JS, Blot WJ, Li JY, Forman D, Fraumeni JF Jr. Risk factors for stomach cancer in sixty-five Chinese counties. Cancer Epidemiol Biomarkers Prev. 1992 Jan-Feb; 1(2):113-8. |
| 10. Li Q, Li XZ, Wang T, Zhou LW, Feng HQ, Gao L, Pei JR, Lin C, Jiang CX. Selenoprotein P and Yunnan endemic sudden cardiac death—an ecological study. Biol Trace Elem Res. 2013 Jan; 151(1):14-7. |
| 11. Riemersma RA, Oliver M, Elton RA, Alfthan G, Vartiainen E, Salo M, Rubba P, Mancini M, Georgi H, Vuilleumier JP, et al. Plasma antioxidants and coronary heart disease: vitamins C and E, and selenium. Eur J Clin Nutr. 1990 Feb; 44(2):143-50. |
| 12. Wang M, Yu Q. Association between blood heavy metal concentrations and skin cancer in the National Health and Nutrition Examination Survey, 2011-2018. Environ Sci Pollut Res Int. 2023 Oct; 30(50):108681-108693. |
| 13. Xie B, Wang J, Zhang J, Chen M. Dietary and serum selenium in coronary heart disease and all-cause mortality: An international perspective. Asia Pac J Clin Nutr. 2020;29(4): 827-838. |
| 14. Yu SY, Chu YJ, Gong XL, Hou C, Li WG, Gong HM, Xie JR. Regional variation of cancer mortality incidence and its relation to selenium levels in China. Biol Trace Elem Res. 1985 Feb; 7(1):21-9. |
| **(D) Same cohort used as in other publication (9 studies)** |
| **Dongfeng-Tongji cohort** |
| 1. Wang C, Zhong G, Liu C, Hong S, Guan X, Xiao Y, Fu M, Zhou Y, You Y, Wu T, Zhao H, Wang Y, Chen S, Zhang Y, Wang C, Guo H. DNA methylation aging signatures of multiple metals exposure and their mediation effects in metal-associated mortality: Evidence from the Dongfeng-Tongji cohort study. J Hazard Mater. 2024 Mar 5; 465:133200. |
| **Eastern Finland Heart Survey** |
| 2. Salonen JT, Alfthan G, Huttunen JK, Pikkarainen J, Puska P. Serum selenium and risk of death in coronary disease. Duodecim. 1983; 99(6):388-95. Finnish. |
| 3. Salonen JT, Salonen R, Penttilä I, Herranen J, Jauhiainen M, Kantola M, Lappeteläinen R, Mäenpää PH, Alfthan G, Puska P. Serum fatty acids, apolipoproteins, selenium and vitamin antioxidants and the risk of death from coronary artery disease. Am J Cardiol. 1985 Aug 1; 56(4):226-31. |
| **National Health and Nutrition Examination Survey (NHANES)** |
| 4. Bleys J, Navas-Acien A, Guallar E. Serum selenium levels and all-cause, cancer, and cardiovascular mortality among US adults. Arch Intern Med. 2008 Feb 25; 168(4):404-10. |
| 5. Eaton CB, Abdul Baki AR, Waring ME, Roberts MB, Lu B. The association of low selenium and renal insufficiency with coronary heart disease and all-cause mortality: NHANES III follow-up study. Atherosclerosis. 2010 Oct; 212(2):689-94. |
| 6. Zhao S, Wang S, Yang X, Shen L. Dose-response relationship between multiple trace elements and risk of all-cause mortality: a prospective cohort study. Front Nutr. 2023;10:1205537. https://doi.org/10.3389/fnut.2023.1205537. |
| 7. RaYe X, Xu T, Yang L, Hu X, Xie X, Lan G, Lu X, Huang Z, Wang T, Wu J, Lan J, Zhang Q, Zhan Z, Guo Y, Xie X. Association between plasma metal exposure and health span in very elderly adults: a prospective cohort study with mixture statistical approach. BMC Geriatr. 2024 May 1;24(1):388. |
| 8. Chu PL, Hsiao CC, Su TC, Wang C, Lin CY. Urinary glyphosate, selenium status, and their impact on mortality: Evidence from NHANES 2013-2018. Ecotoxicol Environ Saf. 2025 Mar 1;292:117989. |
| **Women's Health and Aging Study (WHAS) I and II** |
| 9. Ray AL, Semba RD, Walston J, Ferrucci L, Cappola AR, Ricks MO, Xue QL, Fried LP. Low serum selenium and total carotenoids predict mortality among older women living in the community: the women's health and aging studies. J Nutr. 2006 Jan; 136(1):172-6. |
| **(E) No reported cutoff of selenium category (1 study)** |
| 1. Marniemi J, Järvisalo J, Toikka T, Räihä I, Ahotupa M, Sourander L. Blood vitamins, mineral elements and inflammation markers as risk factors of vascular and non-vascular disease mortality in an elderly population. Int J Epidemiol. 1998 Oct;27(5):799-807. |
| **(F) No reported case number for each selenium category (5 studies)** |
| 1. Kornitzer M, Valente F, De Bacquer D, Neve J, De Backer G. Serum selenium and cancer mortality: a nested case-control study within an age- and sex-stratified sample of the Belgian adult population. Eur J Clin Nutr. 2004 Jan;58(1):98-104. |
| 2. Salonen JT, Alfthan G, Huttunen JK, Pikkarainen J, Puska P. Association between cardiovascular death and myocardial infarction and serum selenium in a matched-pair longitudinal study. Lancet. 1982 Jul 24;2(8291):175-9. |
| 3. Virtamo J, Valkeila E, Alfthan G, Punsar S, Huttunen JK, Karvonen MJ. Serum selenium and the risk of coronary heart disease and stroke. Am J Epidemiol. 1985 Aug;122(2):276-82. |
| 4. Xing X, Xu M, Yang L, Shao C, Wang Y, Qi M, Niu X, Gao D. Association of selenium and cadmium with heart failure and mortality based on the National Health and Nutrition Examination Survey. J Hum Nutr Diet. 2023 Aug;36(4):1496-1506. |
| 5. Xiao S, Wang Z, Zuo R, Zhou Y, Wang Z, Chen T, Liu N. Association of serum five heavy metals level with all-cause and cause-specific mortality: a large population-based cohort study. J Environ Sci Health A Tox Hazard Subst Environ Eng. 2024;59(3):130-154. |

# **Appendix Table 6.** Quality of studies evaluated for cohort studies according to a modified Newcastle-Ottawa-Scale

| **First author, year** | **Representativeness of the exposed cohort** | **Selection of the non-exposed cohort** | **Ascertainment of exposure** | **Demonstration that outcome of interest was not present at start of study** | **Comparability of cohorts on the basis of the design or analysis^a^** | **Assessment of outcome** | **Was follow-up long enough for outcomes to occur^b^** | **Adequacy of follow up of cohorts^c^** | **Total score** |
| --- | --- | --- | --- | --- | --- | --- | --- | --- | --- |
| Kok, 2001  CVD mortality | 1 | 1 | 1 | 1 | 2 | 1 | 1 | 0 | 8 |
| Kok, 2001  Cancer mortality | 1 | 1 | 1 | 1 | 2 | 1 | 1 | 0 | 8 |
| Kilander, 2001 | 1 | 1 | 1 | 1 | 0 | 1 | 1 | 1 | 7 |
| Wei, 2004 | 0 | 1 | 1 | 1 | 2 | 1 | 1 | 1 | 8 |
| Akbaraly, 2005 | 1 | 1 | 1 | 1 | 2 | 0 | 1 | 0 | 7 |
| Walston, 2006 | 1 | 1 | 1 | 1 | 2 | 1 | 1 | 0 | 8 |
| González, 2007 | 1 | 1 | 1 | 1 | 2 | 0 | 1 | 1 | 8 |
| Lauretani, 2008 | 1 | 1 | 1 | 1 | 0 | 1 | 1 | 1 | 7 |
| Bates, 2011 | 1 | 1 | 1 | 1 | 2 | 1 | 1 | 0 | 8 |
| Suadicani, 2012 | 1 | 1 | 1 | 1 | 0 | 1 | 1 | 1 | 7 |
| Goyal, 2013 | 1 | 1 | 1 | 1 | 2 | 1 | 1 | 1 | 9 |
| Alehagen, 2016 | 1 | 1 | 1 | 1 | 0 | 1 | 1 | 0 | 6 |
| Giovannini, 2018 | 1 | 1 | 1 | 1 | 0 | 1 | 1 | 1 | 7 |
| Schomburg, 2019 | 1 | 1 | 1 | 1 | 2 | 1 | 1 | 1 | 9 |
| Li, 2020 | 1 | 1 | 1 | 1 | 2 | 1 | 1 | 0 | 8 |
| Shi, 2021 | 1 | 1 | 1 | 1 | 2 | 1 | 1 | 1 | 9 |
| Al-Mubarak, 2022 | 0 | 1 | 1 | 1 | 2 | 1 | 1 | 0 | 7 |
| Schöttker, 2024 | 1 | 1 | 1 | 1 | 2 | 1 | 1 | 0 | 8 |
| Li, 2024 | 1 | 1 | 1 | 1 | 0 | 1 | 1 | 0 | 6 |
| Jiang, 2024 | 1 | 1 | 1 | 1 | 0 | 1 | 1 | 0 | 6 |

^a^ For the comparability, one point was allocated to the studies controlling for age, sex, body mass index or equivalent, and smoking, and the second point was allocated to the studies controlling for any additional factors.

^b^ For follow-up time of the outcome, one point was allocated to studies with at least 4-year follow-up.

^c^ For the adequacy of follow-up of cohorts, one point was allocated to studies with at least 80% follow-up.


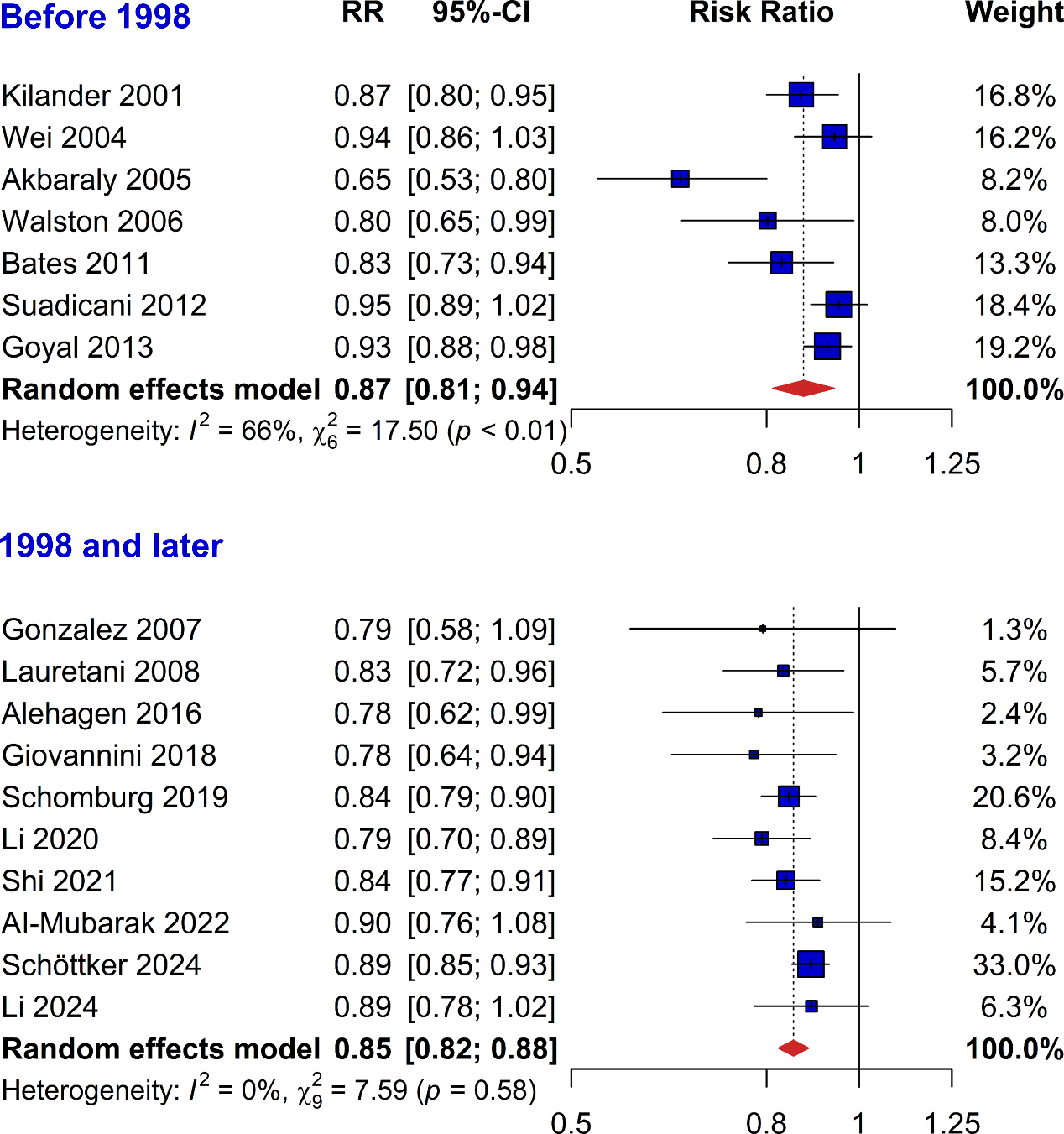


# **Appendix Figure 1.** Subgroup meta-analyses by recruitment years of the studies for the association of plasma/serum selenium or selenoprotein P concentration (risk ratios per 1 standard deviation) with all-cause mortality

Abbreviations: 95%-CI, 95% confidence interval; RR, risk ratio.


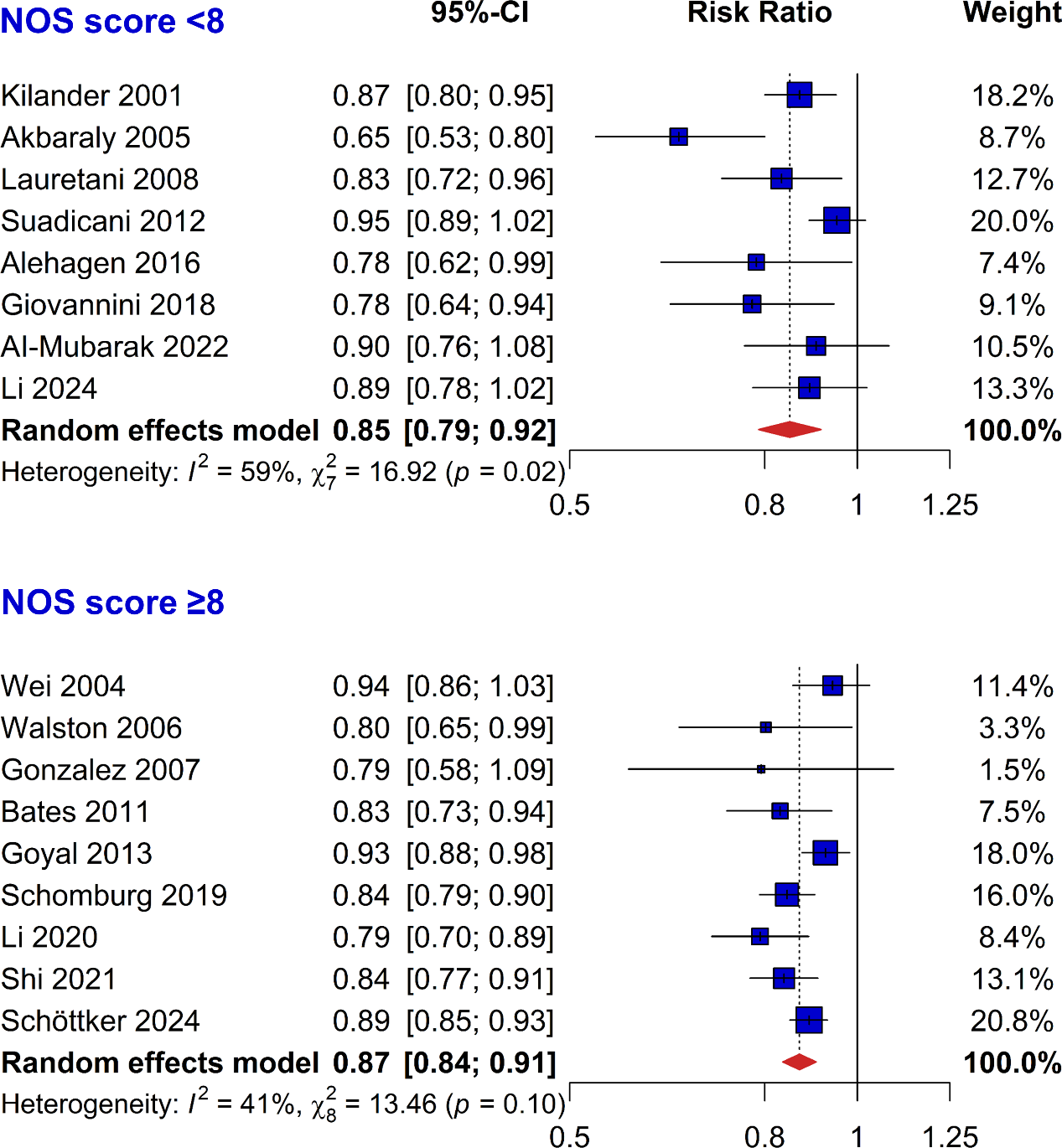


# **Appendix Figure 2.** Subgroup meta-analyses by the modified Newcastle-Ottawa scale scores for the association of plasma/serum selenium or selenoprotein P concentration (risk ratios per 1 standard deviation) with all-cause mortality

Abbreviations: 95%-CI, 95% confidence interval; RR, risk ratio; NOS, modified Newcastle-Ottawa scale.


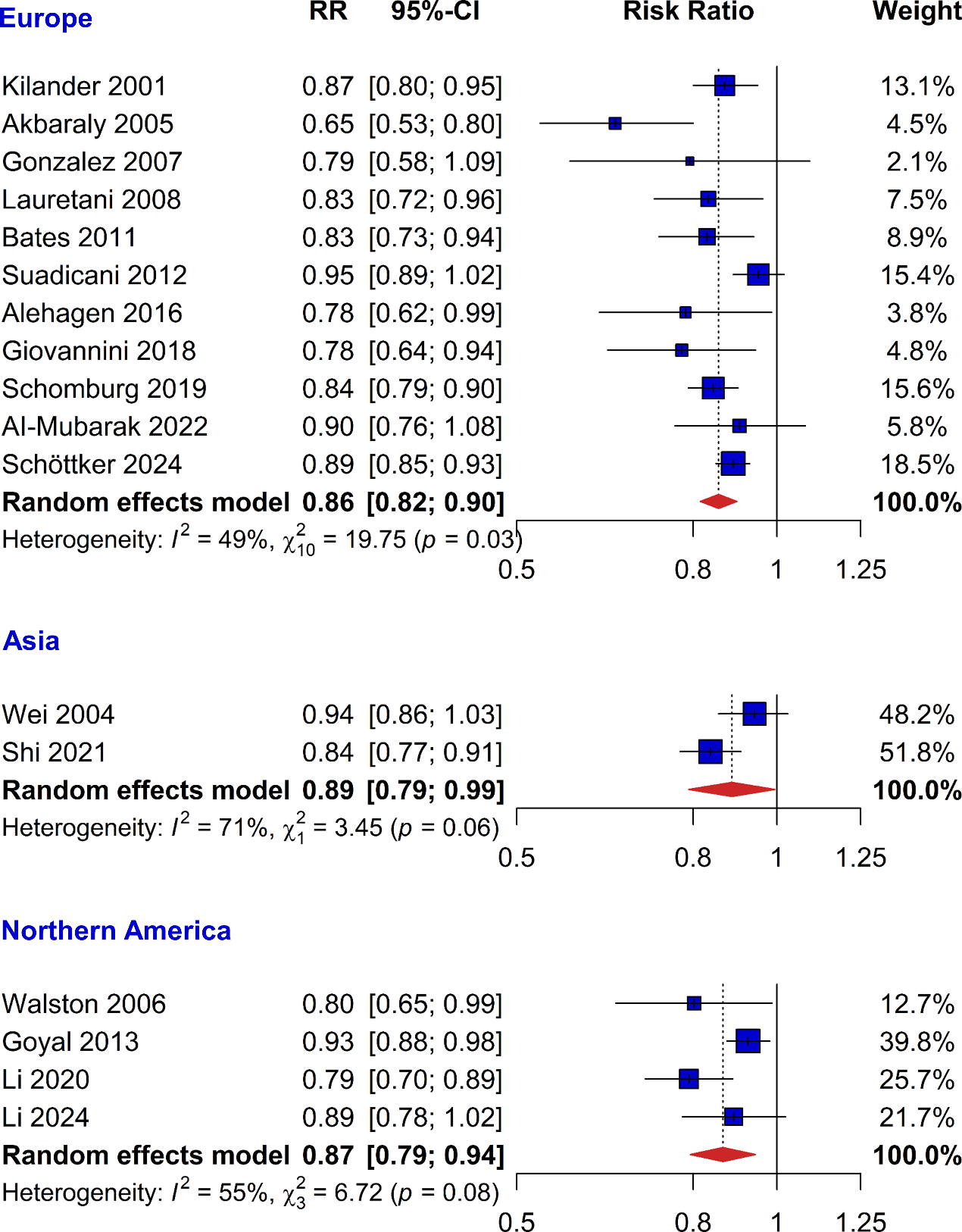


# **Appendix Figure 3.** Subgroup meta-analyses by region for the association of plasma/serum selenium or selenoprotein P concentrations (risk ratios per 1 standard deviation) with all-cause mortality

Abbreviations: 95%-CI, 95% confidence interval; RR, risk ratio.


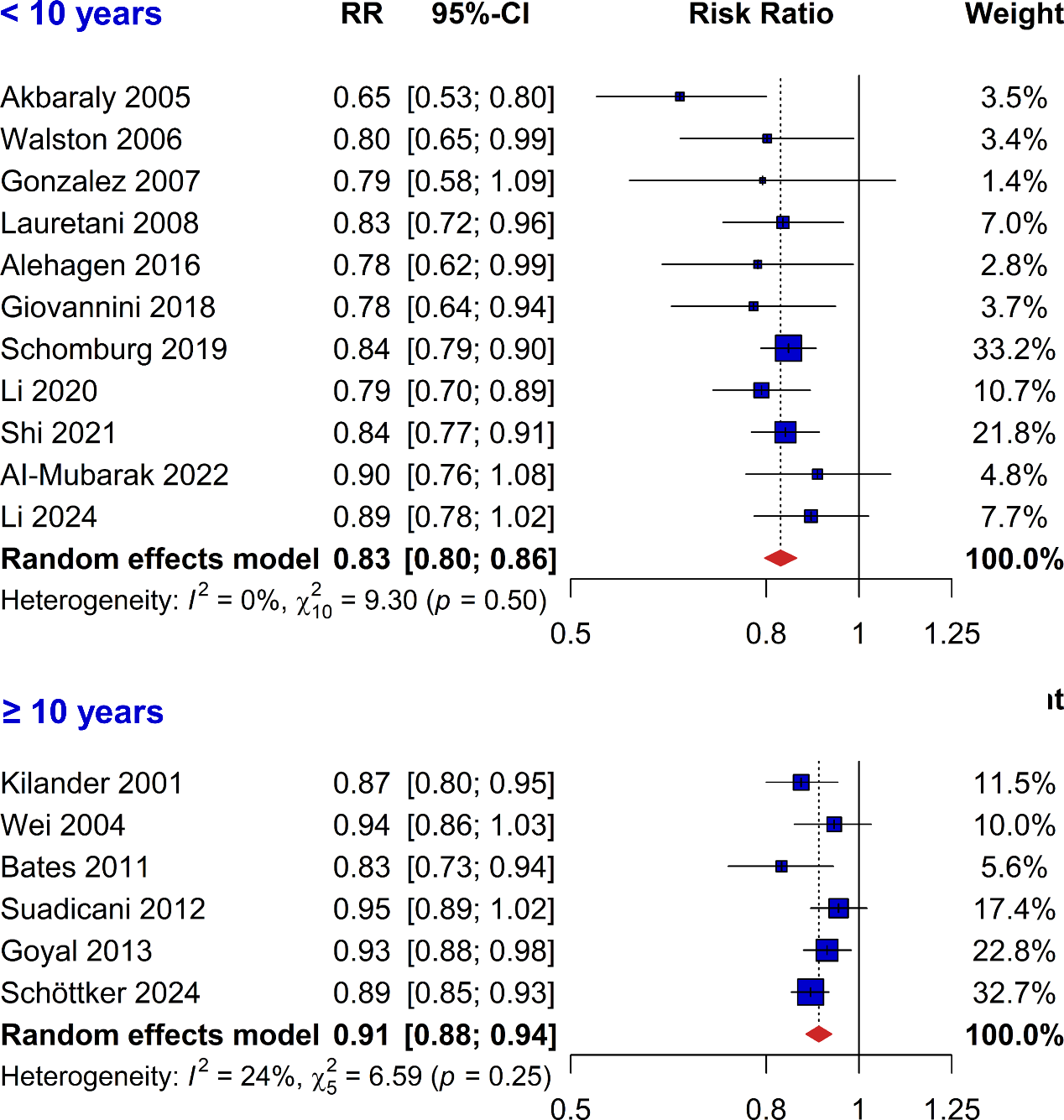


# **Appendix Figure 4.** Subgroup meta-analyses by follow-up time for the association of plasma/serum selenium or selenoprotein P concentrations (risk ratios per 1 standard deviation) with all-cause mortality

Abbreviations: 95%-CI, 95% confidence interval; RR, risk ratio.


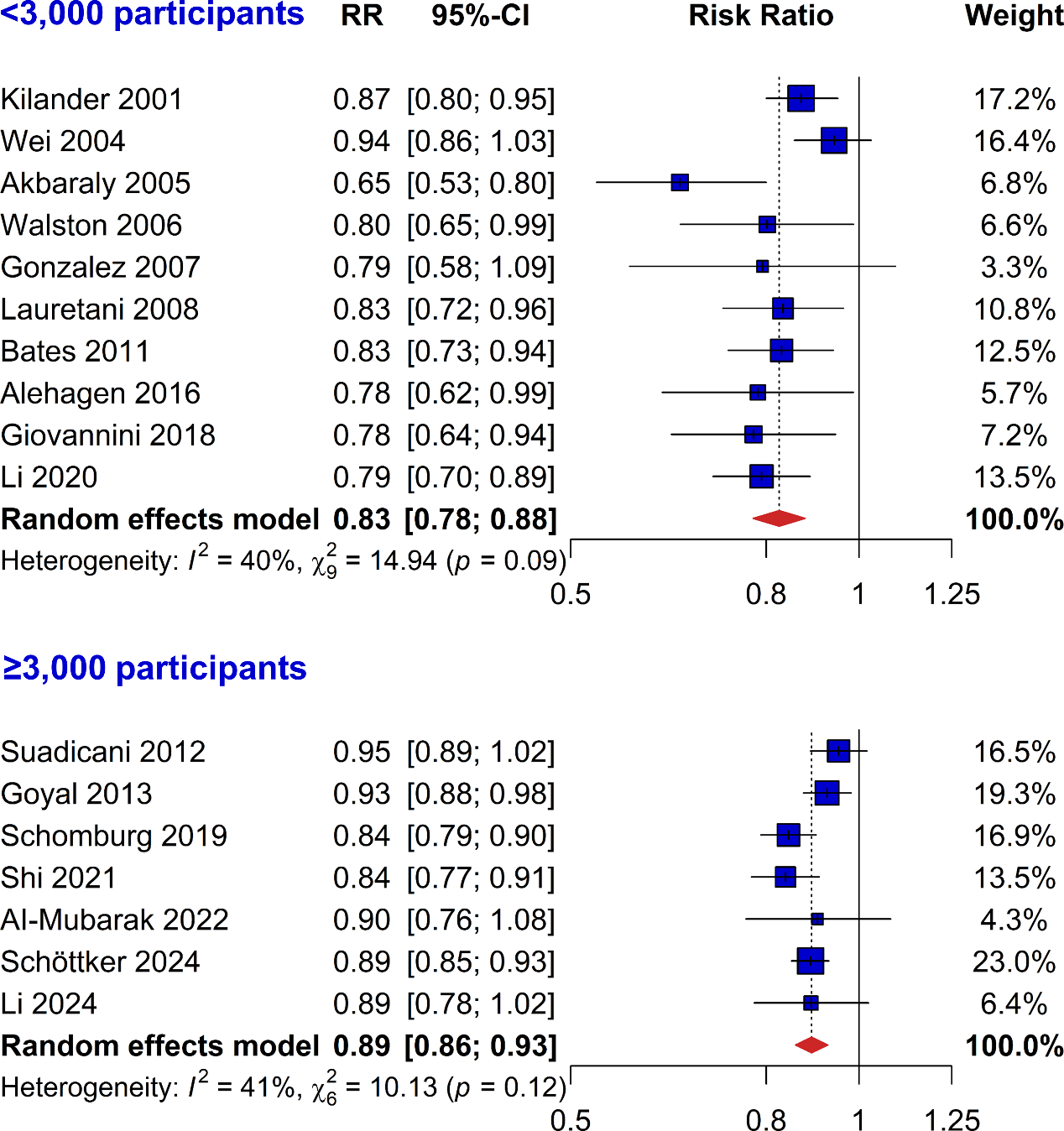


# **Appendix Figure 5.** Subgroup meta-analyses by sample size for the association of plasma/serum selenium or selenoprotein P concentrations (risk ratios per 1 standard deviation) with all-cause mortality

Abbreviations: 95%-CI, 95% confidence interval; RR, risk ratio.


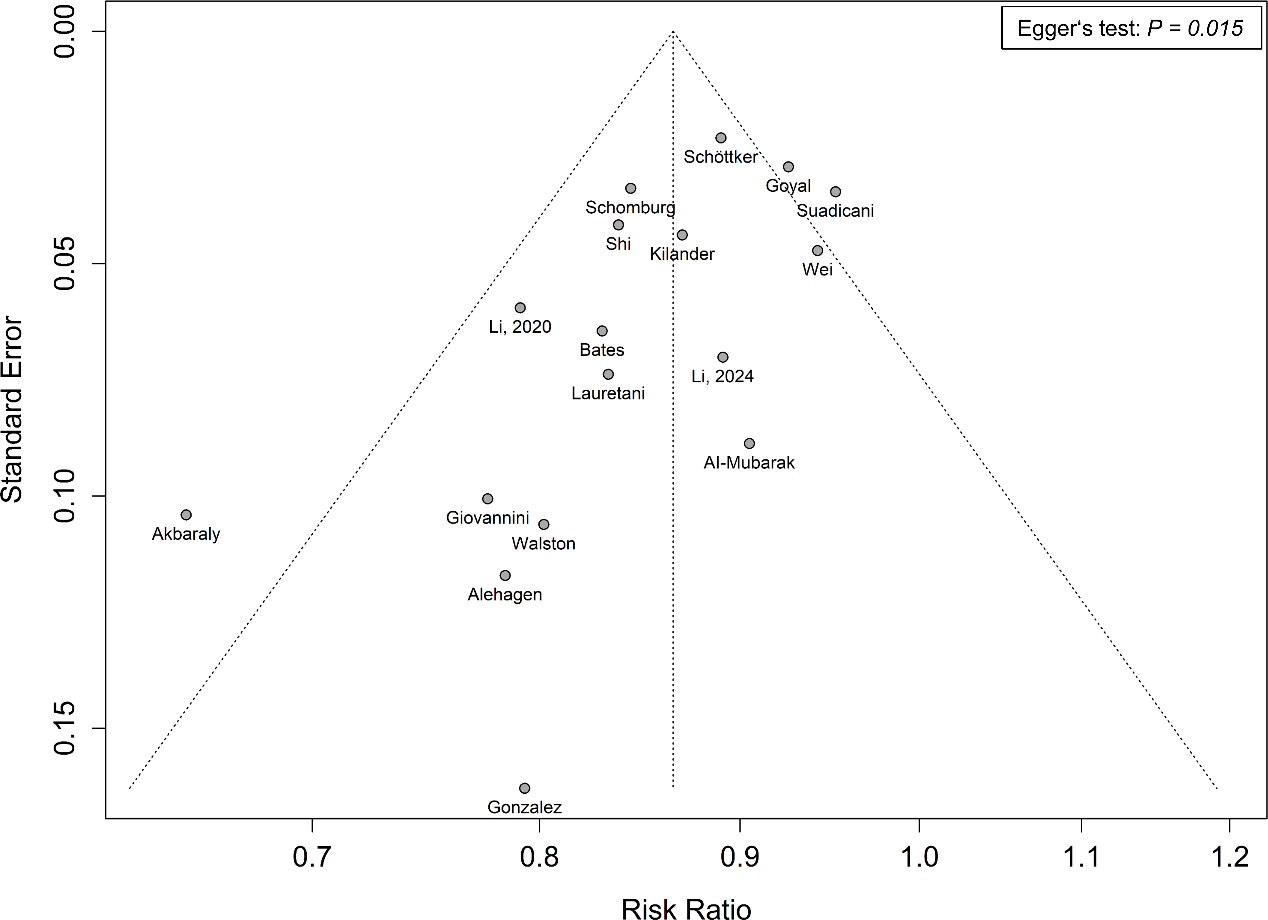


# **Appendix Figure 6.** Funnel plot for the meta-analysis on the association of selenium status with all-cause mortality


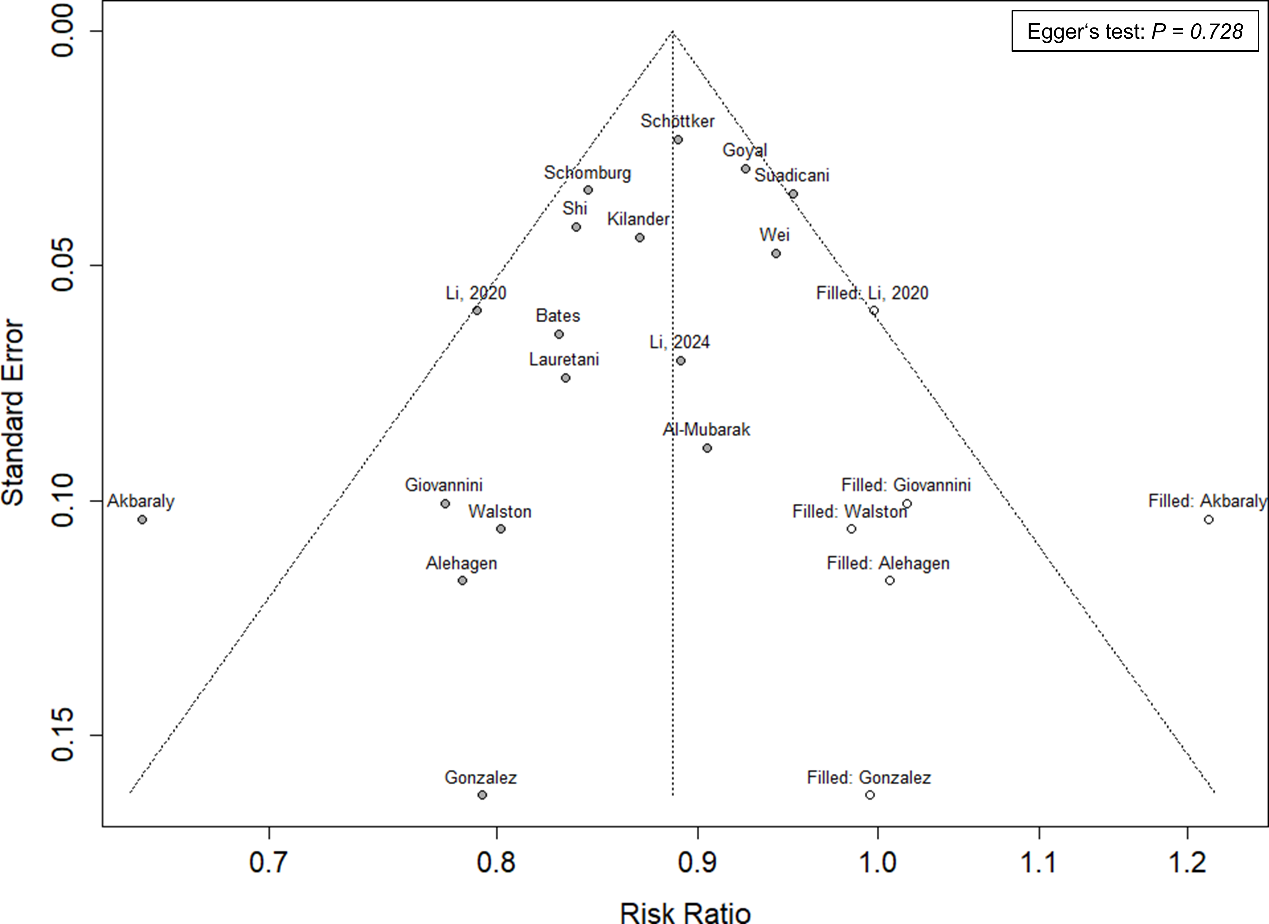


# **Appendix Figure 7.** Funnel plot for the meta-analysis on the association of selenium status with all-cause mortality, including filled studies using trim and fill method

Note: Imputing the potentially unpublished 6 studies with the trim and fill method resulted in a weaker but still statistically significant risk estimate (RR, 95%CI: 0.89 [0.85-0.92] instead of 0.87 [0.83-0.90].


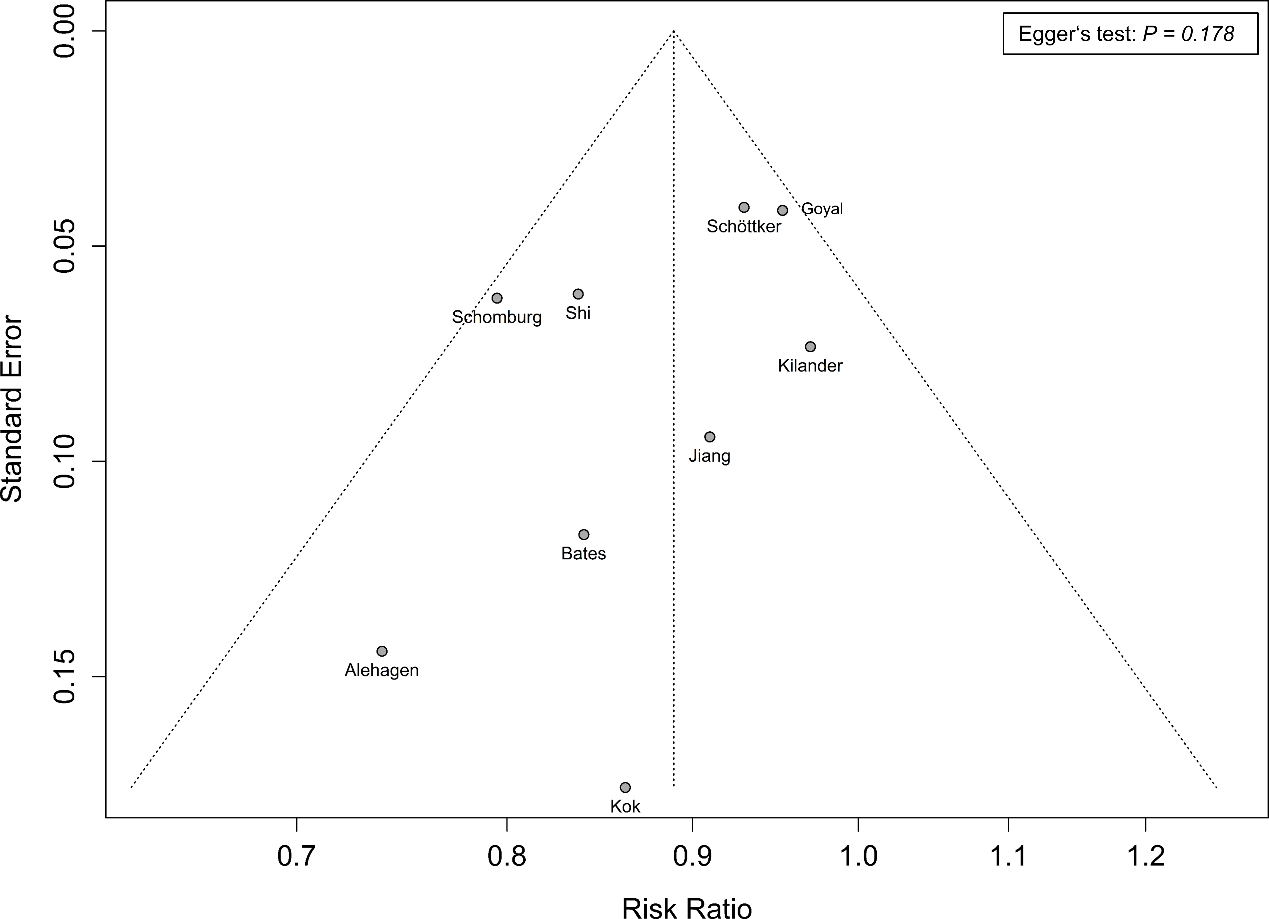


# **Appendix Figure 8** Funnel plot for the meta-analysis on the association of selenium status with cardiovascular mortality


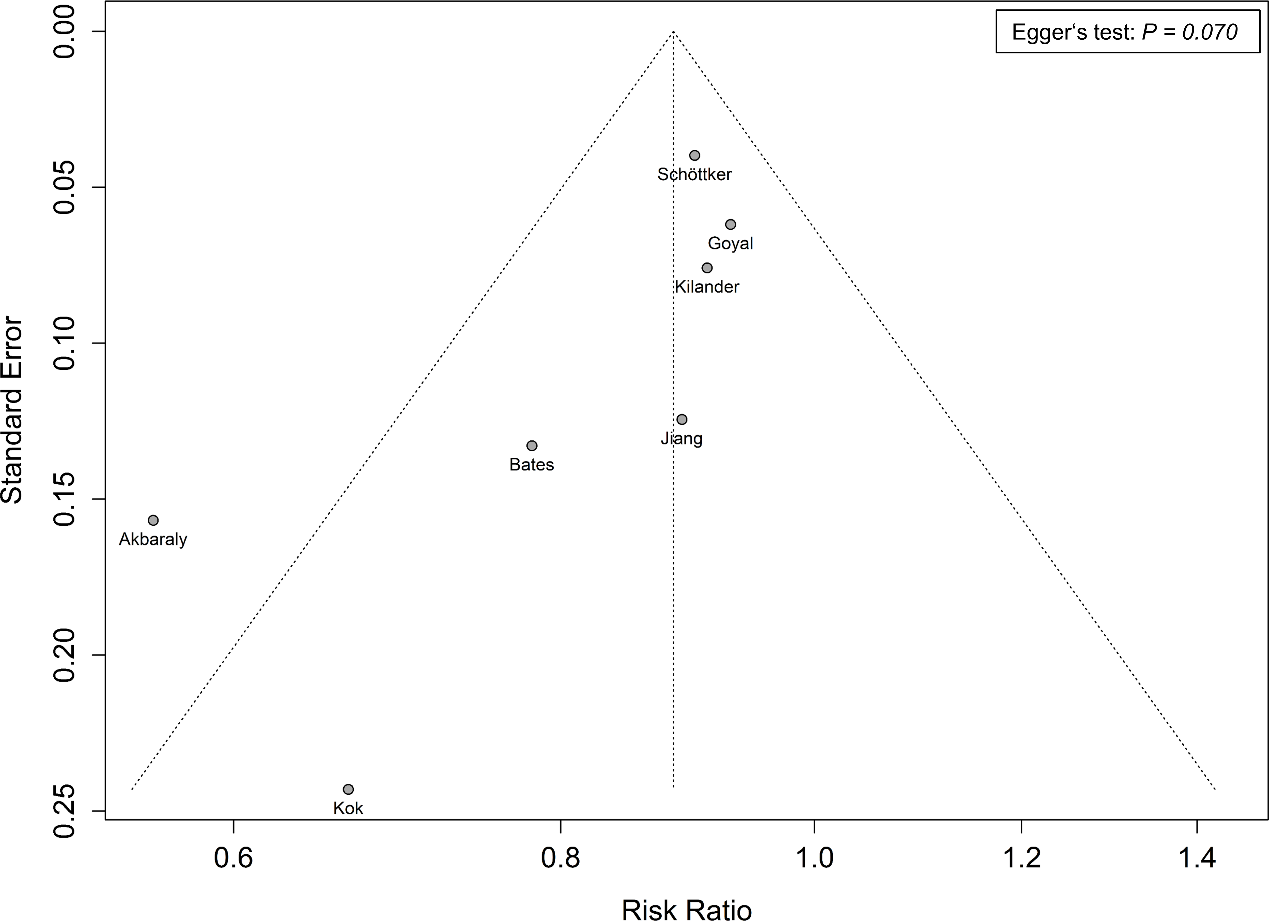


# **Appendix Figure 9.** Funnel plot for the meta-analysis on the association of selenium status with cancer mortality
